# A multifunctional system for genome editing and large-scale interspecies gene transfer

**DOI:** 10.1038/s41467-022-30843-1

**Published:** 2022-06-14

**Authors:** Marc Teufel, Carlo A. Klein, Maurice Mager, Patrick Sobetzko

**Affiliations:** Philipps Universität Marburg, Synthetic Microbiology Center Marburg (SYNMIKRO), Marburg, 35043 Germany

**Keywords:** Synthetic biology, Genetic engineering, CRISPR-Cas9 genome editing

## Abstract

CRISPR SWAPnDROP extends the limits of genome editing to large-scale in-vivo DNA transfer between bacterial species. Its modular platform approach facilitates species specific adaptation to confer genome editing in various species. In this study, we show the implementation of the CRISPR SWAPnDROP concept for the model organism *Escherichia coli*, the fast growing *Vibrio natriegens* and the plant pathogen *Dickeya dadantii*. We demonstrate the excision, transfer and integration of large chromosomal regions between *E. coli*, *V. natriegens* and *D. dadantii* without size-limiting intermediate DNA extraction. CRISPR SWAPnDROP also provides common genome editing approaches comprising scarless, marker-free, iterative and parallel insertions and deletions. The modular character facilitates DNA library applications, and recycling of standardized parts. Its multi-color scarless co-selection system significantly improves editing efficiency and provides visual quality controls throughout the assembly and editing process.

## Abstract (web summary)

The need for diverse chromosomal modifications in biotechnology, synthetic biology and basic research requires the development of new technologies. Here the authors present CRISPR SWAPnDROP, which extends the limits of genome editing to large-scale in-vivo DNA transfer between bacterial species.

## Introduction

In recent years, the relevance of genome editing in bacteria rapidly increased in basic research, biotechnology and synthetic biology. Novel technologies like CRISPR/Cas9 and large scale DNA synthesis brought genome editing within reach of a broad scientific community. However, large-size modifications, incompatibility of individual tools as well as high-throughput systems are still challenging.

The basic steps of genome editing in bacteria is the introduction of exogenous template DNA in the form of plasmids, ssDNA or dsDNA and the subsequent integration into the chromosome via homologous recombination. Homologous recombination is a very inefficient process, even when improved with recombination systems like *λ*RED. Different strategies have been developed to overcome time-consuming screening for edited cells, e.g. the integration of antibiotic resistance markers or metabolic markers^1–4^. However, the number of possible edits in one cell is limited by the number of available markers as only one edit per marker is possible. In addition, the introduction of additional marker genes into the chromosome can cause undesired interference with adjacent transcription units^5,6^. To circumvent the problem of marker limitation, site-specific recombinases such as Cre- and FLP recombinase are introduced to remove the selection marker after chromosomal integration^7,8^. This adds an additional step to the editing process and leaves active recombination sites in the genome, which eventually limits the application of this approach even with the use of alternative recombination sites^9^. Another strategy to avoid selection markers and other scars is oligo-mediated allelic replacement (OMAR). Short single-stranded (ss) DNA are incorporated into the genome by an Okazaki-like allelic-replacement event at the replication fork and facilitate point mutations, small deletions and insertions^10^. Automation and cyclizing of this method allow genome modifications of several genes^11^. However, OMAR is limited by the size of ssDNA oligos used and therefore larger edits are not possible.

For larger scarless genome editing, counter-selection methods can be applied. Such methods rely on efficient counter-selectors like the meganucleases I-SceI and I-CreI, which introduce double-strand breaks at a specific site of 18 and 22 bp in length^12,13^. The double-strand break leads to cell death of non-edited cells. This strongly enriches the viable population for successfully edited cells. However, the approach requires an additional classical editing step, in which the meganucleases’ target site is first integrated into the chromosome at the site of interest.

The discovery of CRISPR/Cas9 abolished the initial integration of a specific restriction site. Similar to a meganuclease, Cas9 is an endonuclease. In contrast to a meganuclease, Cas9 has no DNA sequence specificity. Specificity is mediated by a guide RNA (gRNA) complementary to the target sequence. Possible target sites are only limited by the protospacer adjacent motive (PAM) sequence (5′-NGG-3′), which is required to be located upstream of the target sequence. Due to its simplicity, Cas9 counter-selection is widely used. Plasmid systems harbouring an inducible Cas9 and a recombination system were designed to select for gene deletions, point mutations and short insertions^14,15^. These systems depend on the transformation of synthetic oligonucleotides or linear double-stranded template DNA for homologous recombination and are therefore not suitable for large-size insertion. To overcome size limitations, REXER provides the template DNA on an episomal replicon^16^. Inserts of up to 100 kb are excised in vivo by CRISPR/Cas9 to facilitate *λ*RED recombination. Selection occurs via positive and negative selection markers, which leads to scars at the integration site and requires a previous integration of the first selection-marker set.

For modularization and increased flexibility of CRISPR/Cas9-based methods, modular cloning techniques like Golden Gate Cloning have been employed for sgRNA arrays^17,18^. Furthermore, joining of insert and homology arms as well as the preparation of the locus-specific protospacer require an additional cloning step favouring the concept of modular cloning. Independent of the CRISPR/Cas9 methods, general approaches for modular cloning were developed to assemble larger constructs from small standardized parts, e.g. MoClo, MoCloFlex, Golden Mutagenesis and Marburg Collection^19–22^. However, modular cloning for CRISPR/Cas9 editing has not yet been systematically implemented. The flexibility of modular systems comes at a price. The sequence integrity of the parts from verified plasmid-born DNA libraries is highly stable and needs no additional sequencing after assembly.

Here we present CRISPR SWAPnDROP, a versatile genome-editing system for bacteria. Following a modular concept, CRISPR SWAPnDROP includes a scarless and marker-free system for large-scale insertions, deletions and in vivo chromosomal DNA transfer between strains and even species. For compatibility with automated genome editing, it is capable of multiplexing and iterative genome modifications. Its multi-colour selection system efficiently avoids errors in system assembly and provides a scarless co-selection system for increased editing efficiency.

## Results

### Concept

**Fig. 1:**
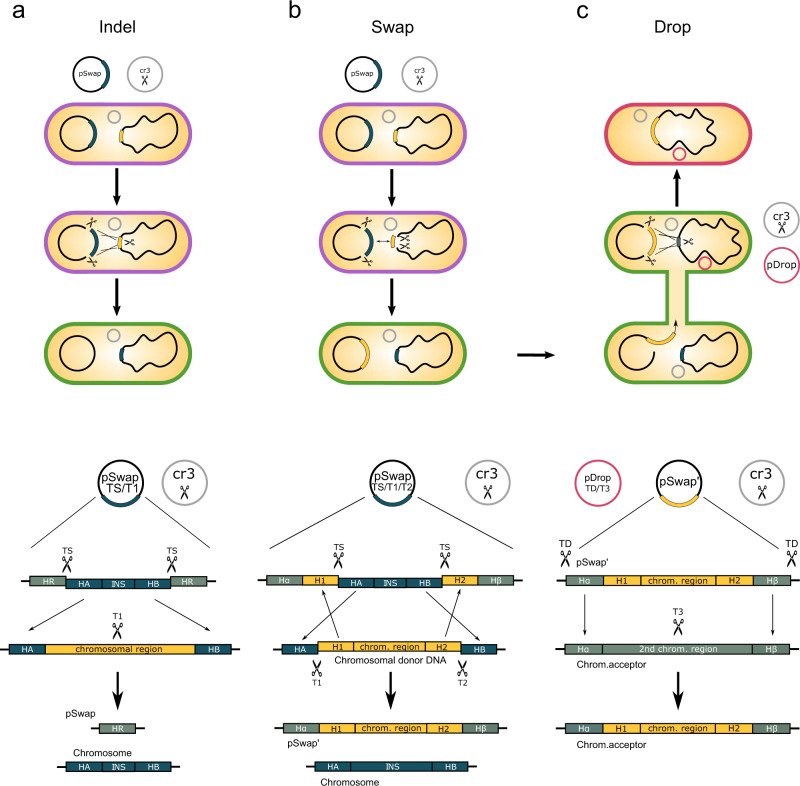
Overview and mechanism of CRISPR SWAPnDROP. CRISPR SWAPnDROP is a genome editing system based on CRISPR/Cas9 counter-selection, homologous recombination and a multi-colour scarless co-selection. It facilitates scarless insertions and deletions (Indel) (**a**) and is capable of transferring chromosomal DNA between organisms (**b**, **c**). The pSwap plasmid harbours the template for homologous recombination including the homologous regions (HA, HB, HR, H1*α*, H2*β*) and the insert DNA (INS), the sgRNA (TS) expression cassettes for the excision of the double-stranded donor template and for the chromosomal counter-selection (T1, T2). The excision is catalysed by CRISPR/Cas9 (Scissors), expressed from the helper plasmid cr3 together with the genes for efficient homologous recombination. For the transfer of chromosomal regions, chromosomal donor DNA (yellow) is additionally excised (T1, T2). The two regions are swapped resulting in a pSwap plasmid loaded with a chromosomal region of interest (pSwap'). The loaded pSwap' plasmid can then be conjugated to a cell harbouring cr3 and the second helper plasmid pDrop, which catalyses the excision of the donor DNA on pSwap' (TD) and the subsequent integration into the acceptor chromosome via counter-selection (T3). Cell border colour represents the multi-colour co-selection system. Cells change their colour from purple to green and from green to red after the Indel/Swap and Drop events, respectively.

DNA synthesis technologies and modular cloning approaches allow for the assembly of large DNA fragments at a scale of bacterial chromosomes. Such large fragments play a role in biotechnology for complex pathway assembly or tailored organism design. Moreover, in synthetic biology and basic chromosome research, rearranged or even completely synthetic chromosomes receive increasing attention^16,23,24^. Such approaches, however, require reliable and versatile handling of large DNA fragments and DNA libraries. We have developed CRISPR SWAPnDROP to meet these requirements. CRISPR SWAPnDROP is based on homologous recombination, CRISPR/Cas9 counter-selection and a scarless multi-colour co-selection system. CRISPR SWAPnDROP provides a framework for the assembly of large genomic sequences, the rearrangement of chromosomal parts as well as the sequence transfer between strains and even to other organisms (see Fig. 1). Furthermore, it comes with a full set of editing tools for convenient iterative and parallel scarless chromosomal deletions and insertions. Supplementary Table 5 provides an overview of the whole workflow regarding days, tasks and expenditure of time.

CRISPR SWAPnDROP is based on homologous recombination of linear double-stranded DNA. It allows for insertion and deletions as well as transfer of large DNA fragments between strains or species (Fig. 1). For insertions and deletions (Indel) two flanking homologies (HA, HB) are used for chromosomal integration of the insert (INS). The fragment HA-INS-HB is released from a plasmid inside the cell by restriction with Cas9. Furthermore, Cas9 is used as a counter-selector using locus-specific sgRNAs expressed from the same plasmid. For the transfer of DNA between cells, chromosomal DNA is loaded onto the plasmid by another set of homologies (H1, H2) matching the flanks of the chromosomal fragment (Swap). Fragment excision and plasmid opening is mediated by Cas9 and sgRNAs specific for the flanks of the chromosomal fragment and the plasmid. After loading the fragment onto the plasmid, it is transferred to another cell via RP4 conjugation or plasmid transformation. In the new host, in analogy to the insertion process, the fragment is released by flanking Cas9 restriction and another pair of homologies (H*α*, H*β*) located at the edges of the fragment confer recombination (Drop). The editing process is supported by a colour system based on the deoxyviolacein pathway and the *mScarlet* gene that provides positive feedback of the experimental state in each step of editing by changing colours (Fig. 1a). Cells harbouring the correct assembled pSwap plasmid produce a purple colour. Cells, which successfully performed Cas9 restriction and homologous recombination during the Indel/Swap and the Drop steps produce a green and red colour, respectively. For CRISPR SWAPnDROP to function properly it is central that recombination and Cas9 restriction works efficiently. Although the CRISPR SWAPnDROP concept described above is generally applicable, it is apparently not possible to provide a static system that will be functional in many species. Given the diversity of species, central components like origins of replication, resistance cassettes, recombination systems or individual promoters and RBS need to be adapted to the individual species. Therefore, we have designed CRISPR SWAPnDROP in a highly modular fashion to allow for efficient combinatorial screenings for the implementation in new species. In this study, we show the implementation process for the model organism *Escherichia coli*, the fast-growing marine bacterium *Vibrio natriegens* and the plant pathogen *Dickeya dadantii*. Details about the features, mechanisms, helper plasmids and modularity are provided in the following paragraphs.

### cr3—the CRISPR SWAPnDROP workhorse

The cr3 helper plasmid harbours the enzymes required for genome editing. These enzymes comprise the Cas9 endonuclease and a species-specific recombination system. The plasmid itself is assembled via the MoCloFlex system^20^. With the MoCloFlex system, the assembly of up to 5 components in any orientation, order and composition is possible in one step. This allows the user to adapt the cr3 plasmid to different species if necessary by exchanging parts, e.g. origin of replication, antibiotic resistance or the recombination system. In this study, we show the composition of cr3 for *E. coli* (cr3Ec), *V. natriegens* (cr3Vn) and *D. dadantii* (cr3Dd).

### pSwap—the modular carrier plasmid

The pSwap plasmid represents the variable component of the CRISPR SWAPnDROP system. It harbours all edit-specific parts including insertion fragments, sgRNAs and homologous regions for the integration and transfer of DNA. The assembly is based on modular cloning and confers edit-specific customization of the pSwap plasmid.

### pDrop—the chromosomal transfer helper plasmid

The pDrop plasmid supports the integration of the transferred chromosomal DNA into the new chromosomal locus. It consists of a fixed and a flexible sgRNA, which are responsible for the in vivo excision of the transferred DNA for homologous recombination and the counter-selection for a successful integration, respectively.

### Cas9-based selection and recombination

CRISPR SWAPnDROP can be used for chromosomal modifications such as insertions and deletions (Indels) as well as for the rearrangement and transfer of chromosomal regions between bacterial strains and species (see Fig. 1). For the creation of Indels, the template for homologous recombination is located on the pSwap plasmid consisting of homologous regions HA and HB flanking the desired insert (INS). Induction of the CRISPR/Cas9 and the recombination system leads to the excision of the template and its integration into the chromosome. Recombination of the homologous regions HR, which flank the excised fragment (HA-INS-HB) and have the same nucleotide sequence, recircularize the pSwap plasmid after excision of the template DNA. This is required to maintain plasmid integrity when no loading of the pSwap for DNA transfer is required (see Fig. 1a). Expression of the T1 sgRNA/Cas9 targeting the chromosomal region allows selection for a successful recombination event. Only cells which lost the target site upon integration of the excised fragment survive. For scarless deletions, it is possible to omit the INS fragment leaving only the homologous regions HA and HB. For the transfer of chromosomal regions, the pSwap can also be loaded with chromosomal DNA. In this case, the regions H1 and H2 are homologous to the flanking regions of the chromosomal region to be transferred (see Fig. 1b). Expression of T1 and T2 sgRNA/Cas9 as well as TS sgRNA/Cas9 cause excision of the chromosomal region and the HA-INS-HB fragment, respectively. The recombination system then catalyses the swap of both DNA fragments leading to a loaded pSwap (pSwap’) with chromosomal DNA and a chromosome with a deletion or a substitute fragment. Here, the sgRNAs act as excision and selection tools both on the chromosome and the pSwap plasmid. For dropping the loaded sequence at the desired location, the swap approach can be further extended by an additional set of homologies H*α* and H*β* that flank the region of integration. This is supported by the helper plasmid pDrop expressing sgRNAs TD and T3, which are used for the in vivo excision of the loaded DNA and the selection at the insertion locus, respectively (see Fig. 1c).

### Modular assembly of the pSwap plasmid

**Fig. 2:**
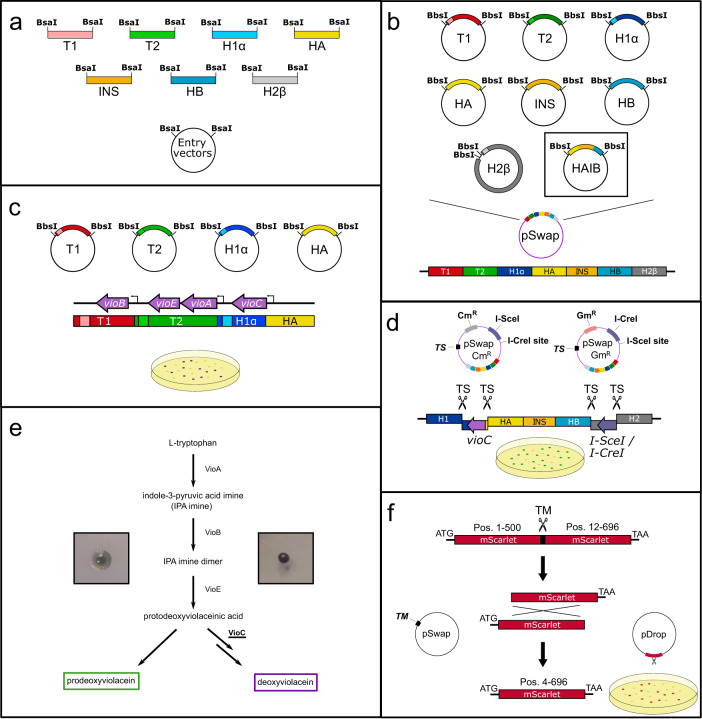
pSwap assembly and recombination overview. CRISPR SWAPnDROP uses a modular assembly system for the pSwap construction. It consists of seven modules (five for scarless integration) harbouring sgRNA expression cassettes for target sites (T1,T2), homologous regions (H*α*, H*β*, H1, H2, HA, HB) and insert DNA (INS). **a** Each of those parts can be cloned into a different entry vector using the type-IIS restriction enzyme BsaI. **b** Each of those seven (five for scarless integration) modules are used to assemble the pSwap plasmid. The assembly takes place in a one-pot, one-step reaction using the type-IIS restriction enzyme BbsI. Therefore, it is possible to combine and recycle different modules for the desired approach. **c** The correct assembly of the pSwap plasmid is ensured by the expression of the biosynthetic pathway of deoxyviolacein. Each of the fragments (T1, T2, H1*α*, HA) harbour parts of the deoxyviolacein expression cassettes (*vioBEAC*). Only if assembled correctly, production of deoxyviolacein is possible. Colonies appear purple after transformation. **d** During linearization of the pSwap plasmid by sgRNA TS and Cas9, the *vioC* gene is removed resulting in the formation of prodeoxyviolacein after successful recombination. Colonies then appear green. In addition, the tool box comprises two pSwap plasmids (Cm^*R*^/Gm^*R*^) harbouring either the *I-SceI* or *I-CreI* meganuclease genes and the opposite recognition site. The removal of the meganuclease genes during recombination allows iterative use of the pSwap plasmids and therefore consecutive genomic edits. **e** Shown is the biosynthetic pathway of L-tryptophan to deoxyviolacein. If the gene (*vioC*) for the conversion of protodeoxyviolacein to deoxyviolacein, which is a purple pigment, is missing, a metabolic shift takes place towards prodeoxyviolacein, which presents a green colour. **f** The pDrop plasmid harbours a partly duplicated, non-functional *mScarlet* gene. During Drop recombination, the pDrop plasmid is cut by the sgRNA TM, which is expressed on the pSwap plasmid and subsequently recombined resulting in a functional *mScarlet* gene. Colonies appear red after successful recombination.

**Fig. 3:**
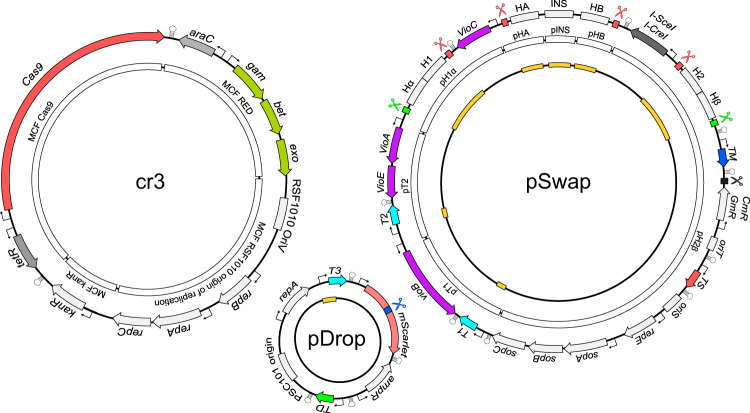
Detailed map of the plasmids cr3Ec, pSwap and pDrop. The outer layer contains relevant features of each plasmid. The white inner layer (if applicable) represents the modular parts prior to assembly using MoCloFlex (MCF) or the CRISPR SWAPnDROP assembly system. The black inner layer (if applicable) shows the location of variable elements (orange) of the plasmids specific for each edit. Cas9 and meganuclease target sites are indicated by scissors. The sgRNA loci and the respective target sites share the same colour.

The pSwap plasmid consists of seven locus-specific parts necessary for genome editing, e.g. homologous regions (HA, HB, H1, H2, H*α*, H*β*), sgRNA expression cassettes for counter-selection and excision (T1, T2) or insert DNA (INS). Each part has its own vector that can be joined into a pSwap plasmid with the desired parts, allowing for the recycling of parts, e.g. the recycling of homologies and sgRNAs to insert different sequence at the same location, or the recycling of the insert at different locations (see Fig. 2a and b). This approach reduces cloning efforts and supports the storage-efficient implementation of a parts library. Moreover, for the pSwap plasmid, assembled from sequenced parts, no additional sequencing is required. The pSwap assembly follows a simple hierarchical topology similar to other cloning systems^19,20^ and is also based on Golden-Gate cloning. Each of the seven modules has its specific level 1 entry vector for cloning the desired parts (see Fig. 2a). In addition, the INS entry vector is level 1 MoClo^19^, MoCloFlex^20^ and Marburg Collection^22^ compatible. Hence, level 1 transcription units build with the MoClo-System or Marburg Collection as well as larger assemblies build with MoCloFlex can be cloned into the INS entry vector. Therefore, already present libraries for these systems can be accessed by CRISPR SWAPnDROP and used for chromosomal integration. Except for the H2, each entry vector contains a high-copy pUC origin of replication, a kanamycin resistance cassette and all contain the *ccdB* and *lacZα* genes for selective cloning^25^. The selective cassette is flanked by two BsaI restriction sites for Golden Gate cloning of the desired DNA fragments. As BsaI is a type-IIS restriction enzyme, cleavage occurs outside of its recognition sequence and therefore corresponding sites are lost upon restriction. This allows cloning in a one-pot, one-step reaction, in which the selective cassette is removed and replaced with the desired DNA fragment. Each of the resulting level 1 vectors contain two additional type-IIS BbsI restriction sites flanking the cloned DNA fragment for the assembly of a level 2 pSwap plasmid. All necessary level 1 modules are cleaved with BbsI and the DNA fragments are assembled via DNA ligase in a one-pot, one-step reaction due to fixed overhangs (see Fig. 2b). In addition to the other level 1 entry vectors, the different versions of the H2 entry vector (Cm^*R*^, Gm^*R*^) contain a single-copy F-origin of replication and a chloramphenicol or gentamycin cassette for the final pSwap. The single-copy H2 plasmid also contains a RP4 origin of transfer for conjugation of large genomic regions. For the scarless integration into the chromosome, HAIB plasmid can be used replacing the modules HA, INS and HB. A standard assembly of pSwap comprises 7 parts. With a growing number of parts in a Golden Gate reaction, the number of correctly assembled plasmids decrease^20^. The selection for a correctly assembled pSwap is guided by co-expression of the deoxyviolacein pathway, whose individual genes are distributed among the parts T1, T2, H1*α* and HA. A successful assembly results in the formation of purple colonies after transformation (see Fig. 2c, e and Supplementary Fig. 1a). This allows the direct transformation of non-cloning strains that naturally exhibit higher frequencies of incorrect clones. Within this study, all selected purple colonies lead to successful edits indicating correct pSwap assemblies. A detailed plasmid map of pSwap is depicted in Fig. 3.

### Design and construction of cr3Ec, cr3Vn and cr3Dd plasmids

The construction of the cr3Ec, cr3Vn and cr3Dd plasmids was carried out using the modular cloning systems MoCloFlex^20^ and the Marburg Collection^22^. The Marburg collection was used to assemble individual transcription units from promoter, RBS, CDS, tag and terminator parts in the library. In addition, the library was extended with newly designed parts. From the Marburg collection, the origin of replication as well as resistance marker, cas9 and recombination transcription units were transferred into position vectors AB, CD, EF and IJ of the MoCloFlex (MCF) system, respectively. The position vectors together with MCF linkers BC, DE, FI and JA allowed us to assemble a set of cr3 variants, each harbouring different combinations of origins of replication, resistance cassettes and induction systems for CRISPR/Cas9 and homologous recombination. The variants were then screened for compatibility with the targeted organism and the other helper plasmids pSwap and pDrop. For the construction of the cr3Ec, the broad host range origin of replication RSF1010 (MCF RSF1010 origin of replication), a kanamycin-resistant cassette (MCF KanR), Cas9 transcription unit (MCF Cas9) and a *λ*RED transcription unit (MCF RED) (see Fig. 3) was assembled. In accordance with Reisch and Prather^14^, the Cas9 transcription unit was assembled using an inducible tetracycline promoter (P_*tet*_), a weak ribosomal binding site, which was integrated as a level 0 part into the Marburg Collection library, the Cas9 coding sequence, the M0051 ssrA degradation tag and the B0015 terminator. For the Cas9 coding sequence, Esp3I, BbsI and BsaI recognition sites were removed by introducing silent mutations to confer compatibility with the Marburg Collection, MoCloFlex and CRISPR SWAPnDROP Golden Gate systems. For efficient homologous recombination in *E. coli* and *V. natriegens*, the *λ*RED system was used. Its transcription unit was assembled using the promoter part harbouring the P_*BAD*_ and *araC* of *E. coli*, the Gam, Beta, Exo coding sequence and the B0015 terminator^22^. For the construction of the cr3Vn, the broad host range RSF1010 as well as a codon-optimized kanamycin resistance gene were combined, which were known to ensure plasmid stability in *V. natriegens*^22^. In earlier studies, phage-derived or native recombination systems were used to confer efficient homologous recombination in *V. natriegens*^26,27^. In this study, we modified the *E. coli* *λ*RED operon by setting it under the control of the native *V. natriegens* P_*BAD*_ promoter to support homologous recombination. The repression was conferred by the native *araC* gene located on the chromosome. For the construction of the cr3Dd, we screened for a species-specific recombination system as no functional recombination system was yet known for *D. dadantii*. This was done by a sequence homology search for known recombination systems in bacteria. In the *D. dadantii* strain Yana2-2^28^, we identified a prophage containing an operon consisting of five genes where two genes showed high amino acid homology to a recombination system present in *Salmonella enterica*. The genes comprised *bet* (89% identity), *exo* (92%) identity. These two genes also showed homology to *bet* and *exo* of *λ*RED (bet: 84% and exo: 86%). The third gene in the operon was identified to be a DNA methyl transferase. Genes four and five were short predicted genes with unknown function. Before transfer of the recombination system to CRISPR SWAPnDROP, we screened for resistance cassettes, origins of replications and induction systems in *D. dadantii* using the modular MoCloFlex approach already applied for *E. coli* and *V. natriegens*. The RSF1010 origin of replication together with the kanamycin resistance cassette of cr3Vn turned out to be also stable in *D. dadantii*. For the induction systems, we chose the cr3Ec arabinose and tetracycline inducible systems. Functionality was verified by mCherry and mVenus fluorescence reporters instead of Cas9 and the recombination system on CR3Dd. Induction and background fluorescence levels were similar to *E. coli*. However, transformation of cr3Dd with Cas9 and the recombination system was not feasible. Replacement of either Cas9 or the recombination system by its fluorescence reporter parts indicated a problem with the recombination system. Therefore, we designed shortened versions of the recombination operon: bet-exo, bet-exo with the methyl transferase and the full operon as control. Again the full operon was not transferable. However, both shorter versions could be transferred. In the next step, we tested induction to see whether toxic levels of induction could occur. For the bet-exo version, no toxic effects were detected. For the bet-exo with methyl transferase, a strong reduction of viable cells was detected for full induction and also with lower levels. During efficiency determination, it turned out that only the tetp-bet-exo version was functional. Hence, the final cr3Dd harbours this recombination cassette.

### Enhanced editing efficiency by multi-colour co-selection

**Fig. 4:**
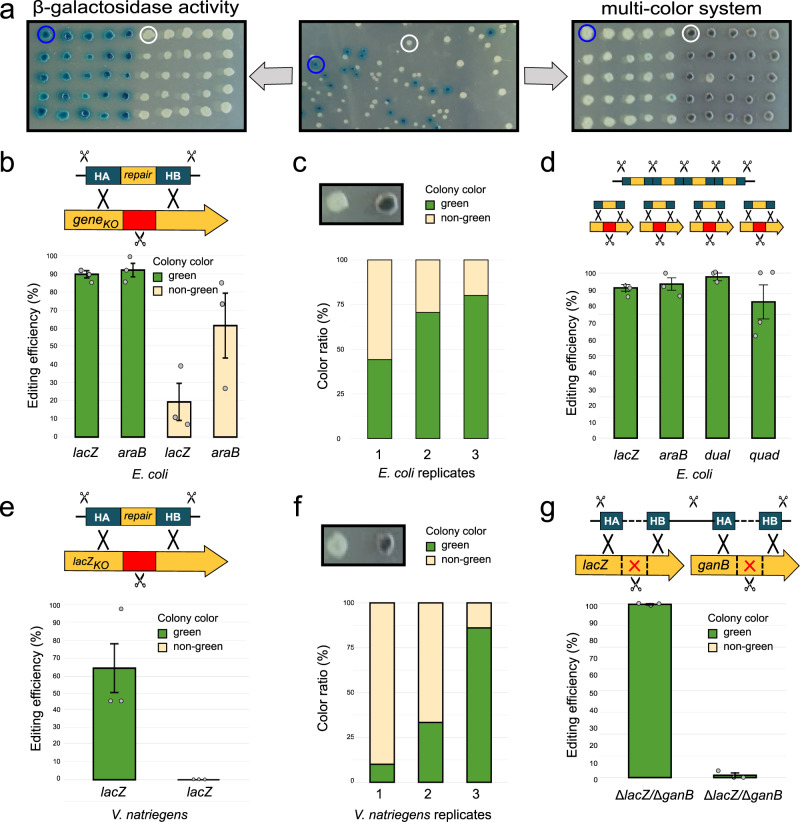
Editing efficiency of CRISPR SWAPnDROP in three species. **a** Test of the multi-colour system by a *β*-galactosidase knockout in *Dickeya dadantii*. In the left panel, *β*-galactosidase positive and negative colonies are picked after editing (centre panel) and grown at 37 ^∘^C on LB agar supplemented with IPTG and X-Gal. The same colonies were also transferred to a second plate and grown on LB at 30 ^∘^C to develop the green colour (right panel). Circles indicate identical colonies on three plates. White colonies in the left and centre panels indicate successful editing. **b** Shown is the editing efficiency of green and non-green colonies for the reconstituted *lacZ* and *araB* genes in *E. coli*. Experiments were carried out in triplicates. 45 green and 45 non-green colonies were tested in total for both *lacZ* and *araB* reconstitution. Data are presented as mean values ± SEM. **c** Shown is the colour distribution of colonies in three different *lacZ* reconstitution experiments. **d** Shown is the editing efficiency of green colonies of single and multiplex editing. Error bars represent the standard error. Experiments were carried out in triplicates, except for the quadruple edit, which was carried out in quadruplicates. For dual editing (*lacZ* and *araB*), 45 colonies and for the quadruple edit (*lacZ*, *araB*, *xylA* and *dapA*), 40 colonies were tested in total. Data are presented as mean values ± SEM. **e** Shown is the editing efficiency of green and non-green colonies for the reconstituted *lacZ* gene in *V. natriegens*. Experiments were carried out in triplicates. In total, 43 green and 32 non-green colonies were tested. Data are presented as mean values ± SEM. **f** Shown is the colour distribution of colonies in three different *lacZ* reconstitution experiments in *V. natriegens*. Error bars represent the standard error. **g** Shown is the editing efficiency of green and non-green colonies for the double knock-out of *lacZ* and *ganB* genes in *D. dadantii*. Experiments were carried out in triplicates. 75 green and 75 non-green colonies were tested in total. Data are presented as mean values ± SEM. Source data are provided as a Source data file.

CRISPR SWAPnDROP enhances editing efficiency by a multi-colour scarless co-selection strategy. This co-selection strategy selects for functional CRISPR/Cas9 and recombination systems, thus decreasing suppressor mutations (see Supplementary Table 6). Consequently, editing efficiency is increased without scars or the trade-off of additional chromosomal markers^29^. The CRISPR SWAPnDROP co-selection is based on the elimination of the *vioC* gene from the pSwap plasmid by CRISPR/Cas9-mediated excision and subsequent recombination upon induction (see Fig. 2d). The lack of *vioC* leads to a metabolic shift towards prodeoxyviolacein and, in turn, to the formation of green colonies (see Figs. 2e and 4a). To assess the gene-editing efficiency of CRISPR SWAPnDROP two *E. coli* genes, *lacZ* and *araB*, were reconstituted and phenotypically tested. For that, two *E. coli* knockout strains were first generated each lacking either a functional *lacZ* or *araB* gene by deleting parts of the corresponding coding sequence and introducing Cas9 target sites. Deleting only parts of the coding sequence renders the genes non-functional. At the same time it ensures that the reconstituting fragment on its own does not restore gene function in the subsequent reconstitution step. In this way, neither the pSwap plasmid containing the reconstituting fragment nor an off-target insertion into another locus causes false-positive results. The genes *lacZ* and *araB* encode for the *β*-galactosidase and ribulokinase, both essential for the lactose and arabinose metabolism, respectively. Therefore, both strains were not able to grow on M9 minimal medium supplemented with lactose or arabinose, respectively. Two pSwap plasmids (pSwap lacZ/pSwap araB) harbouring either the missing part of *lacZ* or *araB* (INS) flanked by corresponding homologous regions HA and HB as well as sgRNAs (T1), specific for the deletion region, were then assembled and transformed into the *lacZ* and *araB* knock-out strains. After induction of the CRISPR/Cas9 and *λ*RED systems, green and non-green colonies were spotted on M9 plates supplemented with either lactose or arabinose. Clones, which scarlessly integrated the missing coding sequence into their genome, were again able to grow on the corresponding saccharide. Clones were verified exemplarily by PCR and Sanger sequencing. For *lacZ* and *araB* reconstitution, green colonies attained an editing efficiency of up to 100% with low variation, while non-green colonies resulted in an editing efficiency of about 20% and 60% with a higher variation, respectively (see Fig. 4b). In addition, the colony colour distribution in different *lacZ* reconstitution experiments revealed a high variation of the green to non-green ratio (see Fig. 4c). In some experiments, the number of green colonies dropped to a few counts on the whole plate whereas editing efficiency of green colony remained high. The frequency of green colonies depends on the timing of suppressor mutant emergence and subsequent outgrowth and is, therefore, random and not controllable. The earlier the mutation during growth, the higher the percentage of suppressors on the plate. Consequently, for some experiments, the total efficiency would drop to a low percentage, due to accumulation of suppressor mutants. However, within the pool of green colonies, the editing efficiency is significantly higher, very stable between replicates and not correlated to the frequency of green colonies. Hence, the multi-colour system of SWAPnDROP increases and stabilizes the expected editing efficiency of CRISPR/Cas9 gene editing. Analogous to the in vivo pSwap cleavage of the green-colour selection for the Indel and Swap recombination, a red-colour selection was implemented for the Drop recombination. The pDrop plasmid harbours a partly duplicated, non-functional *mScarlet* gene, which is cleaved by CRISPR/Cas9 during the Drop. The cleavage is mediated by the sgRNA TM (target mScarlet) located on the pSwap plasmid (see Fig. 2f). Homologous recombination of the overlapping sequences results in a functional *mScarlet* gene and therefore in the formation of red colonies (see Fig. 2f and Supplementary Fig. 1c, d). For a detailed plasmid map of pDrop see Fig. 3.

### Fast iterative genome editing using alternating plasmids

**Fig. 5:**
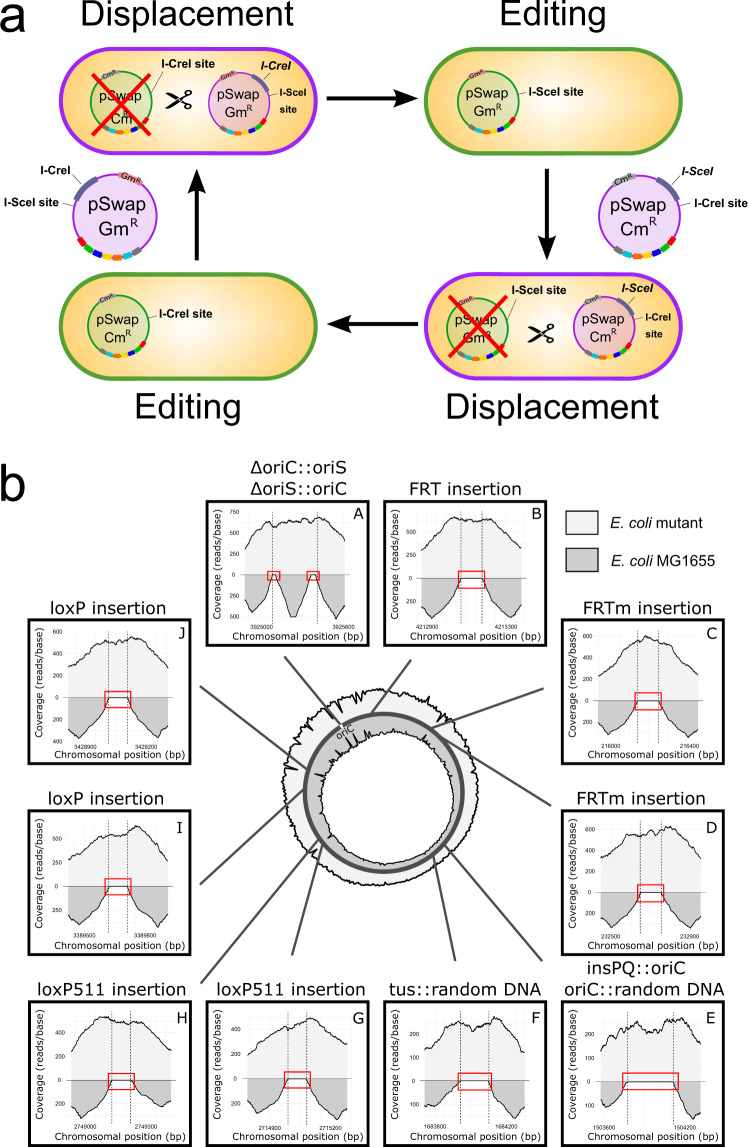
Iterative genome editing with CRISPR SWAPnDROP. **a** The use of the two pSwap plasmids (Cm^*R*^/Gm^*R*^) allows iterative genome editing. Each harbouring either the *I-SceI* or *I-CreI* meganuclease genes and the opposite recognition site I-CreI site or I-SceI site, respectively. During the editing, the meganuclease genes are removed leaving the pSwap plasmid only with the opposite recognition site. Subsequent transformation of the other pSwap plasmid leads to the elimination of the previous plasmid and allows for another round of editing. Cell colours represent the corresponding editing step. **b** Next-generation sequencing of *E. coli* mutant after 12 consecutive edits. Shown is the next-generation sequencing (NGS) coverage of a *E. coli* mutant strain after 12 consecutive edits (light grey) compared to the wild-type *E. coli* MG1655 (dark grey). For the mutant strain, several recombination sites (FRT/loxP) as well as random DNA and origins of replication were inserted into the chromosome using iterative CRISPR SWAPnDROP genome editing. Mutant and wild-type *E. coli* NGS reads were aligned against the mutant reference genome and the sectors of each edited site (I–X) as well as the complete genome coverage (circle) are shown. Reads at all insertion locations (dashed lines) are present for the mutant strain, while no reads are present for the wild-type strain (red rectangle). (I) and (V) show the coverage of a chromosomal location after 2 different edits at the same position. For (I), the native origin of replication *oriC* of *E. coli* was first replaced with the F-plasmid derived oriS origin of replication. oriS was then replaced again with *oriC* while introducing scar sites flanking the oriC. Scar sites were introduced due to using HA, INS and HB plasmids instead of HAIB plasmid. The absence of those flanking scars in the wild-type is highlighted with red rectangles in (I). For (V), *oriC* was first inserted into the *insPQ* locus and then replaced with a random sequence. The absence of the random sequence in the wild-type strain is highlighted with a red square.

The CRISPR SWAPnDROP system comes with two complementary versions of the pSwap plasmid: pSwap Cm^*R*^ and Gm^*R*^. The first harbours a constitutively expressed *I-SceI*, a I-CreI recognition site and a Cm^*R*^ cassette. The second harbours a constitutively expressed *I-CreI*, a I-SceI recognition site and a Gm^*R*^ cassette (see Fig. 5a). During recombination events in the editing process, the meganuclease genes are removed from the pSwap plasmid by CRISPR/Cas9 excision together with the *vioC* gene (see Fig. 2d). The resulting green edited clones carry a pSwap’ plasmid only harbouring the recognition site of the other meganuclease (see Fig. 2d). Transformation of a fresh pSwap plasmid cures the clone from the old pSwap by expression of the meganuclease located on the fresh pSwap and allows for another round of editing (see Fig. 5a). Functionality was tested in *E. coli* MG1655 wild type for 12 consecutive genomic inserts at 10 distinct loci using alternating pSwap Cm^*R*^ and pSwap Gm^*R*^ plasmids. During these 12 edits no problem in plasmid curing or evident functional loss of the editing system was observed. During the functionality test, different recombination sites (FRT/loxP) as well as random DNA and origins of replication (F-plasmid oriS/native *E. coli* oriC) were inserted and replaced throughout the genome of *E. coli* MG1655 (see Fig. 5b). The use of alternating pSwap plasmids facilitates one round of editing every 3 days. This is particularly useful in case of the absence of a suitable PAM site. A new site can be introduced by a first edit in close proximity and the desired edit can be done efficiently in a consecutive edit reverting the first edit and introducing the desired modification. Moreover, the approach not only facilitates and speeds up consecutive rounds of edits, but also makes CRISPR SWAPnDROP compatible with automated editing approaches.

### Multiplex genome editing

To assess the efficiency of CRISPR SWAPnDROP multiplex genome editing, the two *E. coli* genes, *lacZ* and *araB*, were reconstituted simultaneously. In a first step, an *E. coli* strain was generated by two consecutive gene knock-outs of *lacZ* and *araB* via CRISPR SWAPnDROP. Consequently, the strain was unable to grow on M9 plates supplemented with lactose or arabinose. In a second step, a pSwap for parallel repair was assembled. The pSwap plasmid contained the two repair templates (HA*lacZ*-INS*lacZ*-HB*lacZ*-TS-HA*araB*-INS*araB*-HB*araB*) separated by an additional Cas9 excision site (TS) as well as the sgRNAs (T1, T2) targeting the non-functional *lacZ* and *araB* deletion sites (see Supplementary Fig. 2). The repair templates were cloned via Golden Gate cloning in pHAIB using 6 PCR fragments (HA*lacZ*, INS*lacZ*, HB*lacZ*-TS, HA*araB*, INS*araB*, HB*araB*). The additional Cas9 target site (TS) separates the templates for each locus of separate recombination via *λ*RED. After induction of CRISPR/Cas9 and *λ*RED, green colonies were transferred to M9 plates containing either lactose or arabinose. Consequently, only colonies with successfully reconstituted *lacZ* and *araB* genes were able to grow on both plates. The editing efficiency of the dual-repair approach was 98%, which means almost all clones restored their ability to grow on both saccharides. Even though, CRISPR SWAPnDROP is currently not able to generate more than two simultaneous edits due to its limitation of two sgRNAs, we wanted to assess the multiplex editing potential of larger numbers of parallel edits for future extensions of the system by sgRNA arrays^18^. Therefore, two additional genes, *xylA* and *dapA*, were disrupted in the *lacZ* and *araB* double mutant. *xylA* encodes for a xylose isomerase and is essential for the xylose catabolism, while *dapA* encodes for a dihydrodipicolinate synthase, which is essential for cell wall synthesis. Disrupting those genes leads to a strain, which is DAP auxotroph and not able to use xylose as a carbon source. The resulting strain contained the four non-functional genes *lacZ*, *araB*, *xylA* and *dapA*. The lack of two sgRNAs was compensated by introducing the same Cas9 target sites for later repair in *xylA* and *dapA* knockouts as used for the *lacZ* and *araB* knockouts respectively. This allowed us to simultaneously modify four different loci, while only using two sgRNAs (T1, T2). In analogy to the double repair, the pSwap plasmid used for the reconstitution contained the four repair templates, each separated by an additional Cas9 target site (TS). Green colonies were tested on their ability to grow solely on lactose, arabinose and xylose each supplemented with DAP as well as on glucose without DAP. On average, 83% of the tested clones were able to grow on all saccharides as well as without DAP (see Fig. 4d). This shows the high editing efficiency of CRISPR SWAPnDROP even for up to four simultaneous edits and supports future extensions of the system by sgRNA arrays.

### Transfer of large chromosomal regions

**Fig. 6:**
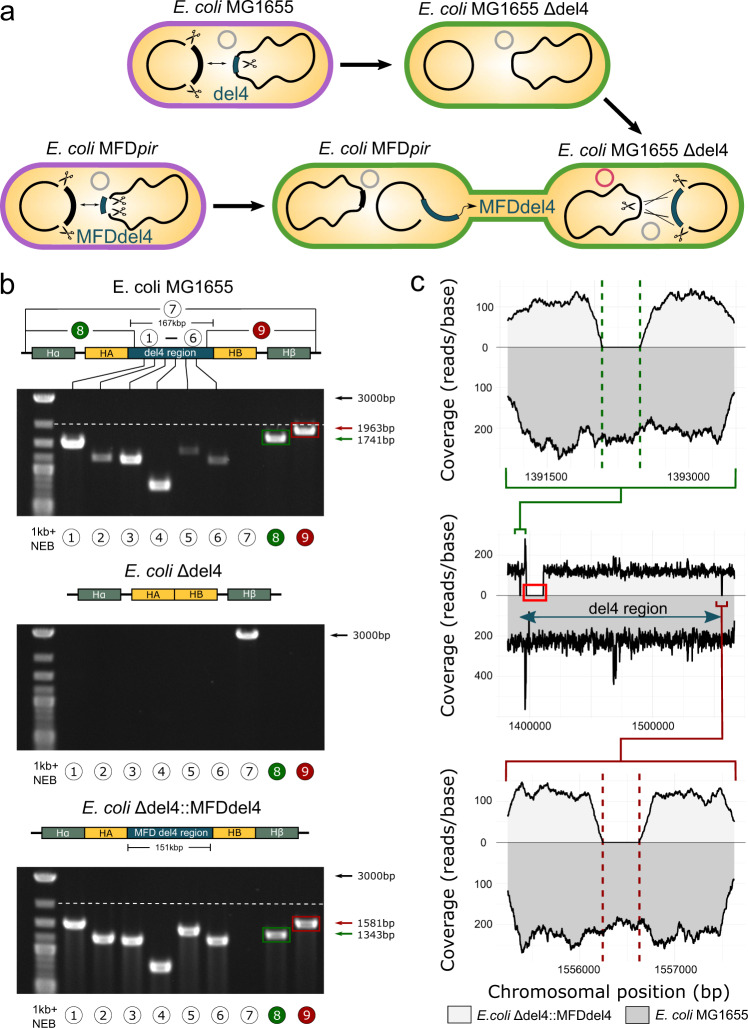
Transfer and integration of a 151 kb chromosomal region via CRISPR SWAPnDROP. **a** Scheme of the deletion of the 167 kb del4 chromosomal region in *E. coli* MG1655 (top). Swap and conjugational transfer of the 151 kb MFDdel4 chromosomal region from *E. coli* MFD*pir* to *E. coli* MG1655 Δdel4 and subsequent integration (bottom). Cell colours represent the corresponding editing step. **b** Shown are agarose gels of PCRs from the integration of a 151 kb chromosomal region in *E. coli*. PCRs were done for *E. coli* MG1655 (acceptor precursor), the acceptor (*E. coli* MG1655 Δdel4) and the edited strain (*E. coli* MG1655 Δdel4::MFDdel4). PCRs “1–6” result in the amplification of fragments of the del4 region evenly distributed over the 167 kb (*E. coli* MG1655) and 151 kb (*E. coli* MFD*pir*), respectively. PCR “7” result in the amplification of a 3000 bp fragment, if the del4 region is missing. PCR “8” and “9” amplify the transition of the del4 region to the adjacent chromosomal regions. Upon integration of the 151 kb MFDdel4 region into the *E. coli* MG1655 Δdel4 chromosome, small regions flanking the 151 kb region were deleted resulting in DNA bands of lower size compared to the wild-type chromosome. This is additionally highlighted with the colours green and red. Dashed line indicates the band size of a 2000 bp DNA fragment for a better comparison. PCR was repeated once with identical results. Chromosomal overviews including homologous regions (HA, HB, H*α*, H*β* of each PCR are shown above each agarose gel. **c** Shown is the next-generation sequencing (NGS) coverage (reads/base) of *E. coli* MG1655 Δdel4::MFDdel4 (light grey) and *E. coli* MG1655 (dark grey) mapped against the *E. coli* MG1655 reference genome. The middle plot shows the coverage of the del4 region. For *E. coli* MG1655 Δdel4::MFDdel4, reads for the 16 kb deletion, only present in the integrated MFDdel4 region, are missing (red rectangle). The upper and lower plots showing the detailed coverage of the del4 flanking regions. Upon integration of the MFDdel4 region, small flanking regions were deleted, shown as missing reads between the dashed lines.

CRISPR/Cas9-mediated excision of double-stranded DNA and homologous recombination generally permit the handling and alteration of large DNA fragments in vivo^16^. CRISPR SWAPnDROP extends the handling of large DNA fragments to a systematic excise and insert. Coupled with the modular assembled pSwap and pDrop plasmids, CRISPR SWAPnDROP provides a tool to rearrange the chromosome and transfer large chromosomal DNA between bacterial strains. As a proof of concept, we transferred and integrated a 151 kb chromosomal fragment from one *E. coli* strain into another (see Fig. 6a). For the sequence to be transferred we chose a chromosomal region (del4), known to cause little impact on strain fitness upon deletion^30^ (see Supplementary Fig. 3). Using CRISPR SWAPnDROP, we first generated a *E. coli* wild-type knockout strain of the del4 region as acceptor for the region. The del4 knock-out avoids possible toxic effects and homology issues due to an additional copy of the del4 region within the *E. coli* chromosome during and after transfer and facilitates transfer validation. For that, *E. coli* MG1655 was first transformed with cr3Ec. A pSwap plasmid was assembled harbouring homologies HA and HB flanking the del4 region as well as a short random insert sequence, which eventually replaced the deleted region. After deletion, the *E. coli* MG1655 Δdel4 was transformed with a pDrop plasmid (pDrop del4) harbouring the sgRNA for selection upon reintegration. A pSwap (pSwap del4) was then assembled and transformed into the *E. coli* RP4 conjugation strain MFD*pir* to load the del4 region onto the plasmid. The del4 region of *E. coli* MFD*pir* is about 151 kb in size and a bit smaller than the deleted *E. coli* MG1655 del4 region due to a ~16 kb deletion within the region. The loaded pSwap’ plasmid was conjugated to *E. coli* MG1655 Δdel4 and the 151 kb region was integrated into the Δdel4 chromosomal region (*E. coli* MG1655 Δdel4::MFDdel4). Colony pcr revealed an integration efficiency of up to 60% and on average around 40% (45 clones were tested). Then, pcr and sanger sequencing was used for verification of each strain. As seen in Fig. 6b, we were able to amplify DNA from the del4 region in *E. coli* MG1655 as well as in *E. coli* MG1655 Δdel4::MFDdel4, while in *E. coli* MG1655 Δdel4 those bands did not appear on the electrophoresis gel. The 3 kb PCR band spanning the del4 region appeared only in *E. coli* MG1655 Δdel4, as for the strains still harbouring the del4 region the PCR product would have been above 167 kb. To distinguish between *E. coli* MG1655 and *E. coli* MG1655 Δdel4::MFDdel4, homologies H*α* and H*β* were chosen to delete small regions flanking the del4 region after integration. DNA bands of lower size from PCRs spanning this small deleted region indicate the integration of the MFDdel4 region into the Δdel4 mutant chromosome (see Fig. 6b). To rule out that specific bands originate from the pSwap’ plasmid, the strain was tested phenotypically and via PCR for the loss of the plasmid. The strain was unable to grow on the corresponding antibiotic and no plasmid specific amplicons were detected. *E. coli* MG1655 Δdel4::MFDdel4 is a chimera of the MG1655 and the *E. coli* MFD*pir* at the del4 locus. To verify the chimeric nature of the strain, we performed next-generation sequencing (NGS) of the *E. coli* MG1655 Δdel4::MFDdel4 (see Fig. 6c). NGS reads of *E. coli* MG1655 Δdel4::MFDdel4 and *E. coli* MG1655 were mapped against the *E. coli* MG1655 reference genome. While for *E. coli* MG1655, the del4 region is 167 kb in size, about 16 kb are missing in the del4 region of *E. coli* MFD*pir*. Consistent with a successful edit, reads of the 16 kb deletion as well as the deleted del4 flanking regions were missing, while reads of the complete MFDdel4 region were present in *E. coli* MG1655 Δdel4::MFDdel4.

### Interspecies gene transfer and genome editing in *V. natriegens*

**Fig. 7:**
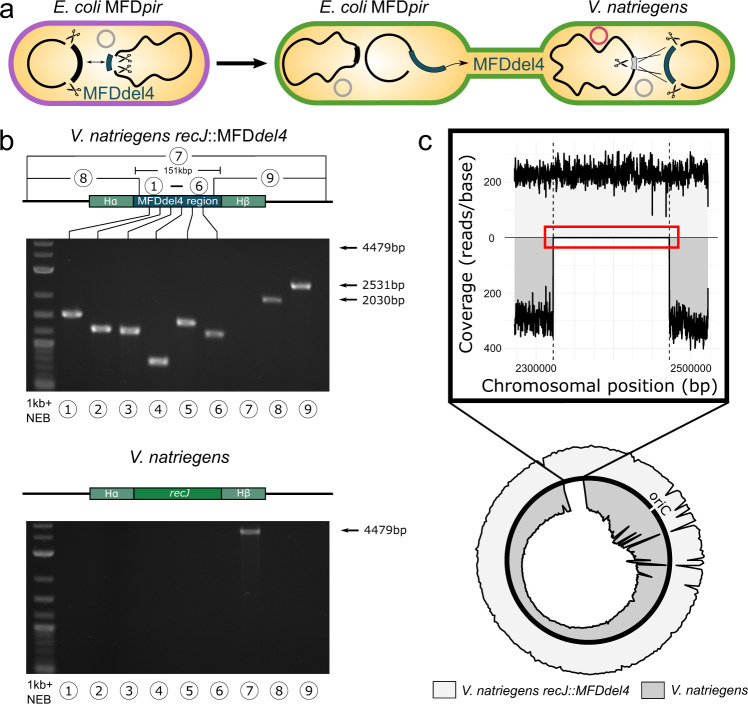
Integration of a 151 kb *E. coli* chromosomal region into the *V. natriegens* chromosome. **a** Scheme of the swap and conjugational transfer of the 151 kb MFDdel4 chromosomal region from *E. coli* to *V. natriegens* and subsequent integration. Cell colours represent the corresponding editing step. **b** Shown are agarose gels of PCRs from the transfer and integration of a 151 kb chromosomal region from *E. coli* into the *recJ* locus of *V. natriegens*. PCRs were done for the acceptor strain (*V. natriegens*) and for the edited strain (*V. natriegensrecJ*::MFDdel4). PCRs “1–6” result in the amplification of evenly distributed fragments of the *E. coli* del4 region within the *V. natriegens* chromosome. PCR “7” results in the amplification of a 4479 bp fragment, if the *recJ* locus is unchanged. PCR “8” and “9” amplify the transition of the del4 region to the adjacent chromosomal regions. PCR was repeated once with identical results. Chromosomal overviews including homologous regions (H*α*, H*β* of each PCR are shown above each agarose gel. **c** Shown is the next-generation sequencing (NGS) coverage of the edited strain *V. natriegensrecJ*::MFDdel4 (light grey) compared to the reference *V. natriegens* strain (dark grey). Both NGS reads were aligned against the edited reference genome (*recJ*::MFDdel4). The sector of the del4 insertion as well as the complete genome coverage (circle) are shown. Reads of the complete del4 insertion (between dashed lines) are present for the edited strain, while no reads are present for the wild-type strain (red rectangle).

**Fig. 8:**
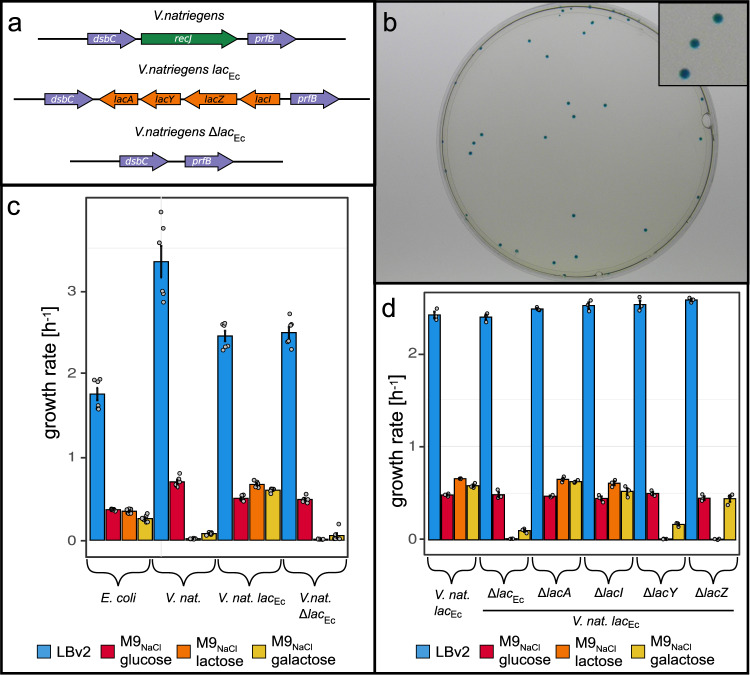
Functionality of the *E. coli* lactose operons in *V. natriegens*. **a** Shown is the gene map of the *V. natriegens* strains tested for lactose metabolism. The native *recJ* gene is replaced by the *E. coli* lac operon in *V. natriegens* lac_*Ec*_. This strain was further edited by the elimination of the lac_*Ec*_ operon resulting in *V. natriegens* Δlac_*Ec*_, which is also a Δ *recJ* knock out. **b** Shown is a picture of *V. natriegens* colonies, harbouring the *E. coli* DH5*α* lac operon, transformed with a plasmid containing the *lacZα* fragment. Cells were plated on LBv2 agar supplemented with IPTG and X-gal. **c** Shown are the growth rates of *V. natriegens* strains and *E. coli* grown in different media and carbon sources. For each sample, six independent replicates were performed. Cells were grown in M9 minimal medium supplemented with either 0.4% glucose (red), lactose (orange) and galactose (yellow) as well as in LB (*E. coli*)/LBv2 medium (*V. natriegens*) (blue) using a microplate reader. Data are presented as mean values ± SEM. **d** Shown are the growth rates of *V*. *natriegenslac*_*EC*_ strain and knockouts of the lac operon and of each gene in the lac operon grown in different media and carbon sources. For each sample, three independent replicates were performed. Cells were grown in M9 minimal medium (+2% NaCl) supplemented with either 0.4% glucose (red), lactose (orange) and galactose (yellow) as well as in LB (*E. coli*)/LBv2 medium (*V. natriegens*) (blue) using a microplate reader. Data are presented as mean values ± SEM. Source data are provided as a Source data file.

The fast-growing gammaproteobacterium *V. natriegens* has the potential to rise as an important organism in biotechnology and molecular biology. In recent years, this organism was made genetically accessible, but still lacks a working CRISPR/Cas9-based gene editing system^31,32^. Using MoCloFlex, we adapted CRISPR SWAPnDROP to *V. natriegens*. In contrast to the pSwap plasmid, the cr3Ec plasmid was not compatible with *V. natriegens*. Hence, we generated a new cr3 version called cr3Vn (see the ‘Design and construction of cr3Ec, cr3Vn and cr3Dd plasmids’ section). To test the system, a pSwap plasmid (pSwap recJ) was assembled containing the selective sgRNA (recJ) and homology arms HA and HB of ~1.2 kb and ~1.4 kb in length, which flank the exonuclease gene *recJ*. Inactivation of *recJ* was shown to enhance natural transformation in other *Vibrio* species^33^. Therefore, the *recJ* locus was chosen with regard to possible future cloning applications. *V. natriegens* was transformed with both cr3Vn and pSwap recJ and purple colonies were used for induction of the Cas9 and *λ*RED genes. Subsequent colony PCR and sanger sequencing revealed *recJ* knock-out clones in 23% of the tested clones (3 out of 13). After showing editing activity of CRISPR SWAPnDROP in *V. natriegens*, we systematically determined the editing efficiency and optimised the induction protocol (see the ‘Methods’ section). In analogy to the quantification of the editing efficiency in *E. coli*, we intended to use a *lacZ* knockout repair approach. For that, we needed a knockout *lacZ* variant of a functional lac operon. As *V. natriegens* has no functional lac operon, a native *lacZ* knockout was no option^34^. Consequently, we planed to transfer the *E. coli* Δ*lacZ* variant used for *E. coli* to *V. natriegens*. First, we transferred the native *E. coli* lac operon to *V. natriegens* to test its functionality. For the transfer, we assembled a pSwap plasmid with homology arms (H1*α*, H2*β*) and sgRNAs (T1, T2) flanking the *E. coli* lac operon (pSwap lacVn). The lac operon was loaded onto the pSwap’, transferred to *V. natriegens* via conjugation and subsequently dropped into the *recJ* locus (see Fig. 8a). Without additional adaptations to the lac operon, *V. natriegens* was able to process X-gal to form blue colonies upon induction with IPTG. In the next step, we transferred the lac operon of the *E. coli* Δ*lacZ* knockout strain to *V. natriegens* harbouring cr3Vn and pDropVn recJ and dropped it into the *recJ* chromosomal locus. For the selective sgRNA of pDropVn recJ, the spacer sequence of the initial *recJ* knockout test was used. For the transfer, we reused pSwap lacVn initially designed to allow for a drop into the *recJ* of *V. natriegens*. Integration was verified by PCR and Sanger sequencing of the transitional region of the insert fragment and the flanking chromosomal region. pSwap plasmid elimination was verified phenotypically by the inability to grow on corresponding antibiotics. With the resulting *V*. *natriegens* *lac*_*Ec*_ Δ*lacZ* strain, repair was performed using the *lacZ* repair pSwap (pSwap lacZ) already used in *E. coli* genome editing efficiency determination. Green and white colonies were examined for their ability to form blue colour upon growth with IPTG and X-gal, which implies a successful editing event. The editing efficiency of the *lacZ* repair in *V. natriegens* was up to 92% and on average around 65% (see Fig. 4e, f). Interestingly, no non-green colonies appeared blue upon growth with IPTG and X-gal indicating an enhanced editing efficiency of green colonies also in *V. natriegens*. In addition, we tested alpha-complementation in *V. natriegens*. In analogy to the previous lac operon transfer, we transferred the lac operon of DH5*α* harbouring only the *lacZω* fragment, capable of alpha-complementation. After transfer and integration into the *recJ* locus by CRISPR SWAPnDROP, the strain was transformed with PICH41308^19^, constitutively expressing the *lacZα* fragment. Blue colonies on plates containing IPTG and X-gal indicated a successful *α*-complementation (see Fig. 8b). We then tested the transfer between species and subsequent integration of a large DNA fragment. For this purpose, we transferred the 151 kb *E. coli* MFDdel4 region to *V. natriegens* and integrated it into the *recJ* chromosomal locus. Analogous to the swap and drop of this region between two *E. coli* strains, we first assembled the same pSwap as for the *E. coli* swap, but exchanged the H*α* and H*β* regions (pSwap del4Vn) to target the *recJ* locus. Afterwards, the MFDdel4 region was loaded onto the pSwap plasmid in *E. coli* and subsequently conjugated to the *V. natriegens* strain harbouring cr3Vn and pDropVn recJ. After dropping the 151 kb region into the *recJ* locus of *V. natriegens*, colony PCR revealed an efficiency of 90%. Plasmid specific PCR and the inability to grow on corresponding antibiotics indicated the elimination of the pSwap’ plasmid. As seen in Fig. 7a, pcr from *V. natriegensrecJ*::MFDdel4 showed DNA bands of the del4 region (see Fig. 7a “1–6”) as well as of the transition between the del4 and the adjacent chromosomal regions (see Fig. 7a “8–9”). In addition, amplification of the *recJ* locus was no longer possible (see Fig. 7a “7”). In order to verify the integrity of the region, we performed next-generation sequencing (NGS). NGS reads of *V. natriegensrecj*::MFDdel4 and *V. natriegens* wild-type (ATCC 14048) were mapped against the *V. natriegensrecj*::MFDdel4 reference genome (see Fig. 7c). Data analysis revealed that indeed reads for the complete 151 kb MFDdel4 region were present in the *recJ* locus of *V. natriegensrecj*::MFDdel4, while they were absent in *V. natriegens* wild-type (see Fig. 7b). After successful transfer of a large region to *V. natriegens*, we transferred the RP4 conjugation system from *E. coli* MFD*pir* to *Vibrio natriegens* into the recJ locus to allow future transfer of large DNA regions from *V. natriegens* to other species (see Supplementary Fig. 4a). In *E. coli* MFD*pir*, RP4 is flanked by two resistance cassettes. These were excluded from the transfer to avoid undesired antibiotic resistances in the final *V. natriegensrecJ*::RP4 strain. After transfer and integration analogous to the MFDdel4 region, chromosomal integration was verified by whole-genome sequencing (see Supplementary Fig. 4b). Conjugation proficiency was tested by transformation of *V. natriegensrecJ*::RP4 with a pSwap plasmid and consecutive conjugational transfer of the plasmid to *E. coli* DH5*α* (see Supplementary Fig. 4c).

### Establishing a lactose/galactose pathway in *V. natriegens*

The *V*. *natriegens* *lac*_*Ec*_ strain created during editing efficiency determination was able to process X-gal. Therefore, we further analysed the lactose metabolism. Growth experiments indicated that *V*. *natriegens* *lac*_*Ec*_ was able to grow on M9 minimal medium with lactose as its sole carbon source (see Fig. 8a, c), even with a slightly higher growth rate (*μ* = 0.67 h^−1^) compared to growth on glucose (*μ* = 0.50 h^−1^). A similar phenomenon was observed for the disaccharide sucrose compared to glucose^34^. Growth rates on rich medium and glucose were slightly reduced compared to wild type. This was also observed in pure Δ*recJ* strains of the initial knockout experiments. Utilization of the second downstream product of lactose degradation, galactose, was strongly improved (*μ* = 0.59 h^−1^) compared to wild type (*μ* = 0.08 h^−1^). In order to exclude secondary mutations in *V*. *natriegenslac*_*Ec*_ as a source of these changes, we removed the lac operon using CRISPR SWAPnDROP. The resulting Δ*lac*_*Ec*_ strain showed the loss of lactose metabolisation and the strongly reduced wild type level of galactose utilization^34^. It retained the slightly reduced growth rates on glucose and rich medium of a *recJ* mutant. Hence, the improved galactose utilization is directly connected to the presence of the *E. coli* lac operon. To link the observation to a single gene in the *lac* operon, we performed single knockouts for each gene (see Fig. 8d). The knockouts of *lacA* and *lacI* showed no deviating phenotype. The *lacZ* knockout resulted in the inability to grow on lactose but had no impact on growth rates on galactose. Finally, the knockout of the lactose transporter *lacY* abolished growth on lactose and restored the low growth rates of *V. natriegens* on galactose. In *E. coli*, *lacY* is known to transport lactose but also has a limited affinity for galactose^35,36^. Hence, the *E. coli lacY* may compensate the suboptimal transport of galactose in *V. natriegens*.

### Interspecies gene transfer and genome editing in *D. dadantii*

**Fig. 9:**
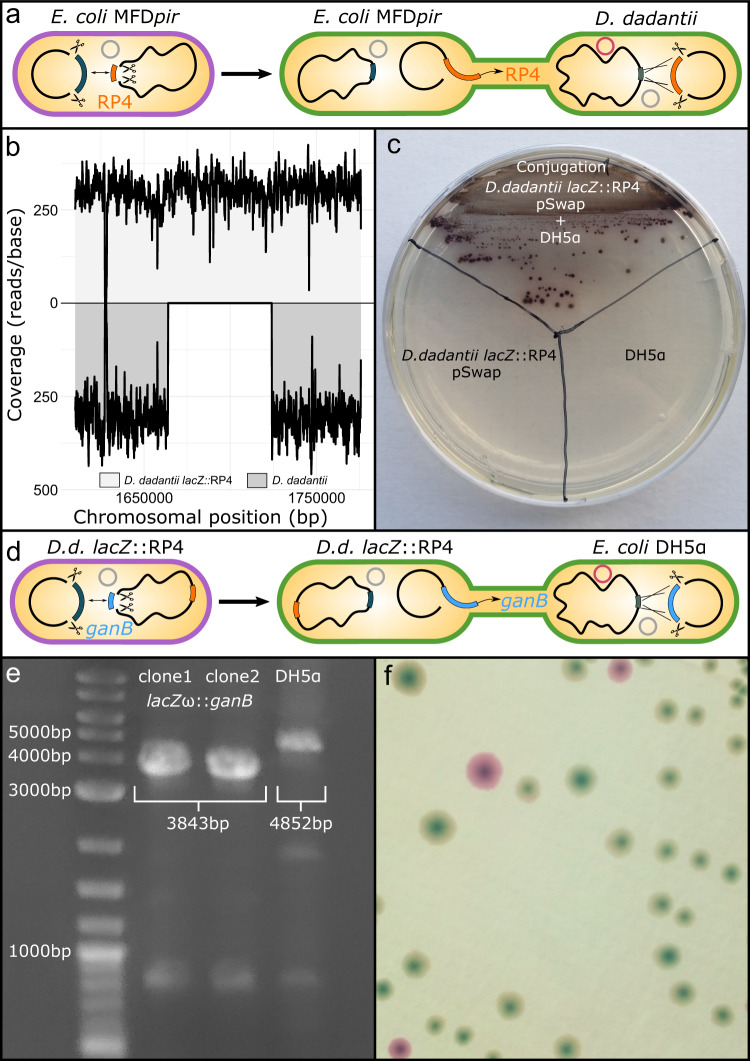
Transfer of the RP4 conjugation system to *D. dadantii* and transfer of the *β*-galactosidase GanB ORF to *E. coli* DH5*α*. **a** Scheme of the swap and conjugational transfer of the RP4 conjugation system from *E. coli* MFDpir to *D. dadantii* and subsequent integration. Cell colours represent the corresponding editing step. **b** Shown is the next-generation sequencing (NGS) coverage (reads/base) of *D. dadantii* *lacZ*::RP4 (light grey) and *D. dadantii* (dark grey) at the RP4 insert locus mapped against the *D. dadantii lacZ*::RP4 reference genome. **c** Conjugation test of pSwap from *D. dadantii* *lacZ*::RP4 to *E. coli* DH5*α*. Controls for donor and acceptor strains are indicated. **d** Scheme of the swap and conjugational transfer of the *ganB* ORF from *D. dadantii* (D.d.)*lacZ*::RP4 to *E. coli* DH5*α* and subsequent integration. Cell colours represent the corresponding editing step. **e** PCR verification of the Drop of *D. dadantii ganB* ORF in the *E. coli* DH5*α* *lacZω* ORF. Two clones after Drop compared to DH5*α*. PCR was repeated once with identical results. **f** Blue colony phenotype of DH5*α* *lacZω*::*ganB* upon induction with IPTG and X-gal. The pDrop plasmid is still present in some colonies of the plated non-selective overnight culture resulting in red colonies.

With the successful implementation of CRISPR SWAPnDROP for *E. coli* and *V. natriegens* we investigated the possibility to establish CRISPR SWAPnDROP for the plant pathogen *Dickeya dadantii* formerly known as *Erwinia chrysanthemi*. It is the causative agent of bacterial stem and root rot affecting potatoes and other crops. For this organism no effective genome editing was feasible due to the lack of a functional recombination system. We identified a phage-derived recombination systems in the *Dickeya dadantii* Yana2-2^28^ strain using a homology-based approach (see ‘Design and construction of cr3Ec, cr3Vn and cr3Dd plasmids’). Furthermore, we tested resistance cassettes, origins of replication and induction systems for functionality in *D. dadantii* to assemble cr3Dd. In a first test, using the newly assembled cr3Dd and a pSwap targeting the *lacZ* locus of *D. dadantii*, we were able to generate a *lacZ* knock-out with high efficiency. As *D. dadantii* harbours two *β*-galactosidase genes, *lacZ* and *ganB*, phenotypic verification analogous to gene editing in *E. coli* and *V. natriegens* was not possible at this stage. We turned to a multiplex approach to knock-out *lacZ* and *ganB* in parallel. All three replicates showed a 100% editing efficiency for green colonies and only 2% for white colonies (see Fig. 4a, g). Hence, editing efficiency was high and the multi-colour system was functional in *D. dadantii*. For the test of gene transfer, we decided to transfer the RP4 conjugation system from *E. coli* MFD*pir* to the *D. dadantii* *lacZ* locus to generate a strain for future transfer of *D. dadantii* genes to other organisms. In analogy to the other two species, homologies for RP4 and the lacZ locus as well as target sites for Cas9 were designed and pSwap/pDrop plasmids were assembled. As for the transfer and insertion of the RP4 conjugation system in *V. natriegens*, the flanking resistance cassettes of the *E. coli* MFD*pir* RP4 region were excluded. In the first step, RP4 was loaded onto the pSwap plasmid using the *E. coli* induction protocol. Green colonies were verified to harbour RP4 by flanking PCRs and conjugated to *D. dadantii* already harbouring cr3Dd and pDrop lacZ. Induction of the Drop system in *D. dadantii* followed the editing protocol for *D. dadantii*. Red colonies were screened for the edit and a positive clone was used for whole-genome sequencing (see Fig. 9a). Activity of the RP4 conjugation system in the *D. dadantii* *lacZ*::RP4 strain was tested by transfer of a pSwap plasmid from *D. dadantii* to *E. coli* DH5*α* (see Fig. 9b). The established conjugation system allows for direct transfer of DNA from *D. dadantii* to other species (see Fig. 9c). To test interspecies chromosomal DNA transfer to *E. coli*, the ORF of the second *β*-galactosidase GanB was selected for scarless transfer into the ORF of *lacZω* of the DH5*α* strain. All parts were designed and assembled in analogy to previous transfers. Swap transfer and drop was performed according to *D. dadantii* and *E. coli* protocols. Transfer was verified phenotypically as well as by PCR and Sanger sequencing (see Fig. 9d–f). As DH5*α* lacks the *α* subunit of LacZ, it is not able to process X-gal to form blue colonies. The replacement of *lacZω* ORF by the *ganB* ORF of *D. dadantii* restored the blue colony phenotype upon IPTG induction with supplemented X-Gal. Compared to the native *lacZ*, X-Gal processing of GanB is lower and takes more time to develop the full-colour intensity. As the delay was not observed in the *D. dadantii* *lacZ* knockout strain only harbouring the GanB *β*-galactosidase, this may be linked to a suboptimal codon usage or activity of the GanB enzyme in *E. coli*.

## Discussion

With CRISPR SWAPnDROP we present a versatile scarless and marker-free genome editing system able to perform indels consecutively or in parallel and transfer chromosomal regions between species independent of size and with high editing efficiencies. In this study, we implement the CRISPR SWAPnDROP concept and test various features for the three species *E. coli*, *V. natriegens* and *D. dadantii*. With its multi-colour co-selection system we introduce an approach to co-selection not relying on chromosomal insertions or other persistent scars and at the same time increasing editing efficiencies and monitoring the assembly process of the modular CRISPR SWAPnDROP system. With its high editing efficiency of above 90% for *E. coli*, CRISPR SWAPnDROP matches the current CRISPR/Cas9-based editing systems^14,15,37^ and integrates desirable features of several systems with high flexibility (see Supplementary Table 7).

CRISPR SWAPnDROP is also capable of multiplexed genome editing. We could show, that even with four parallel edits, the editing efficiency remains well above 80%. This suggests the integration of sgRNAs arrays that have shown to perform up to 10 parallel edits^17,18^ in future extensions.

Its modular design renders integration of these arrays possible without alteration of essential concepts, e.g. by replacing the T1 and T2 modular plasmids by a sgRNA array plasmid. Another aspect of the modular design is the application of libraries for chromosomal integration. In the insert plasmid INS, sequence libraries can be cloned resulting in a broad variety of inserts for a specific location. Furthermore, CRISPR SWAPnDROP is compatible with current cloning systems such as MoClo, MoCloFlex and the Marburg Collection^19,20,22^. This grants access to already established large DNA libraries and facilitates integration of CRISPR SWAPnDROP to present library systems. DNA libraries can be applied in protein modification or pathway optimization and can be directly tested at native chromosomal loci without intermediate plasmid constructs. The modular assembly of the key plasmids cr3 and pSwap simplifies adaptation to other species and facilitates updates with new Cas or recombination systems^38,39^. Using MoCloFlex^20^ and Marburg collection^22^ systems, CRISPR SWAPnDROP was implemented for *E. coli*, *V. natriegens* and *D. dadantii*.

Although the earliest common ancestor of *E. coli* and *V. natriegens* is situated at the phylogenetic level of class (Gammaproteobacteria), only a few parts carrying the resistance cassettes and induction systems needed to be modified to adapt CRISPR SWAPnDROP from *E. coli* to *V. natriegens*. For even more distantly related species, more adaptations may be necessary. In general, for implementing CRISPR SWAPnDROP in a new species, species-specific parts (e.g. promoters, origin of replication and resistance cassettes) need to be chosen. Concerning Cas9 activity, it has been shown that Cas9 is active in all kingdoms of life including animals, fungi, plants, bacteria and archea^40–43^. Therefore, we can assume Cas9 activity in a wide range of species. However, recombination systems are more species-specific. Therefore, for each species, a functional recombination system has to be present. Such recombination systems were found in all kingdoms of life^44–46^ and could be incorporated into CRISPR SWAPnDROP to adapt it to new species. Recombination systems are usually found in species-specific phages/viruses or in the organism itself. In this study, this approach is shown for *Dickeya dadantii* for which no functional heterologous recombination system was known. We identified a phage recombination system in a related strain via sequence analysis and tested different variants of this system using the modular design of cr3. With the resulting cr3Dd and a functional recombination system, we were able to facilitate gene transfer and gene editing in a new species. Furthermore, present recombination systems can be rendered functional in other species by additional expression of missing or incompatible components like single-stranded DNA-binding proteins (SSB)^47^. Using one of the approaches mentioned above, suitable recombination systems should be available for a wide range of species.

The modular design of CRISPR SWAPnDROP is based on the type IIS restriction enzymes BbsI and BsaI. Therefore, homology arms and inserts should be free of these sites to be applied in the system. The recognition sites of the applied restriction enzymes are 6 bases long and therefore occur on average every 4096 bp. For optimal recombination efficiency, homology arms should be about 500 bp in *E. coli*^48^, about 1000 to 3000 bp for *V. natriegens*^27^ and about 1000 bp for *D. dadantii*. However, it is possible to reduce the length to 50 bp for *E. coli* and 200 bp for *V. natriegens* and *D. dadantii*. Therefore, the frequency of a restriction site in a homologous arm is statistically about 1/10 for *V. natriegens* and *D. dadantii* and 1/20 for *E. coli*. Shortening of homology arm, however, may cause a reduction in editing efficiency. Alternatively, restriction sites can be preserved, especially within inserts, by adding a final ligation step to the Golden Gate reaction protocol to religate the preserved sites (see ‘Methods’). It is apparent, that the additional restriction sites reduce assembly efficiency. For cloning of homologies and insert parts, usually one to three fragments are assembled. Within this range, efficiency of Golden Gate cloning is very high, especially with ccdB/lacZ*α* counter-selection used in CRISPR SWAPnDROP. Therefore, a reduced efficiency is no practical burden. For the seven parts of the pSwap assembly, the violacein colour system provides efficient selection for proper clones. Cloning efficiency can be further improved by transformation of a cloning strain instead of the strain of interest.

A general problem of Cas9-based counter-selection is the dependence on PAM sites (NGG) at the locus of interest. Although PAM sites occur on average every 16 bp, in some cases the PAM site might not be situated at the perfect location for a scarless edit. In this case, an additional edit needs to be performed to introduce a PAM site at the desired location. With this new PAM site, the actual scarless edit is possible in a second step. With its ability for stable iterative rounds of genome editing, CRISPR SWAPnDROP facilitates such consecutive edits. In this study, we have shown the stability of the system in 12 consecutive edits. Therefore, the system can be potentially applied in automated cloning approaches^29^.

The application of the meganucleases I-SceI and I-CreI to cure plasmids from the previous iteration, restricts this method to organisms without naturally occurring sites for these meganucleases. However, the length of 18 and 22 bp for I-SceI and I-CreI practically almost excludes occurrences of such sites. The sites occur on average every 68 Gb for I-SceI and every 17 Tb for I-CreI. To illustrate this: Even in the 1000-fold larger human genome no such site is present. However, for the rare case of an occurrence in the genome, the meganucleases and its site on the H2 plasmids can be modified to avoid undesired restriction^49,50^.

The multi-colour selection system introduced in this study improves editing efficiency by selecting out the major part of suppressor mutants that are in previous concepts part of the clones screened for successful edits. Its mechanism provides quality control for the recombination and restriction systems involved in the edit. In general, it relies on parallel plasmid-born edit with a clear phenotype. This concept is realised by Cas9 mediated *vioC* removal and subsequent recombination to reconstitute the pSwap plasmid. Only if Cas9 restriction and recombination are functional green colonies appear on the plate. Therefore, green colonies have a higher chance to yield successful edits and results between replicates are more stable. Like any other selector, the multi-colour system cannot filter all suppressor mutants (e.g. mutations on the target site or sgRNA mutations), therefore editing efficiency is below 100% and residual variance between replicates is detected.

In comparison to *E. coli* and *D. dadantii*, editing efficiency in *V. natriegens* is slightly lower. The decrease in editing efficiency may lie in the efficiency of *E. coli* *λ*RED applied in *V. natriegens*. The recombination system is evolutionary optimized for *E. coli* and apart from the induction system, we made no attempts to further optimize codon usage or RBS strength for *λ*RED in *V. natriegens*. An optimized expression of *λ*RED may improve efficiency even more. Still, screening of two colonies yields a positive clone on average. Therefore CRISPR SWAPnDROP is also highly effective in *V. natriegens*.

CRISPR SWAPnDROP is capable of transferring large chromosomal regions between strains and species. We tested the system in Hfr+ strains. By adding an RP4 plasmid derivative or by triparental mating, the approach can in principle be extended to any strain of interest. However, CRISPR SWAPnDROP can also be used to construct permanent conjugation strains of new species as we have shown for *V. natriegens* and *D. dadantii*. RP4 is a broad-host-range conjugation system, functional in Gram-negative bacteria^51^ and various Gram-positive bacteria^52^. Large scale DNA transfer of CRISPR SWAPnDROP can be applied in synthetic chromosome construction for biotechnology and basic research^23,24,53^. The technical and human effort for the in vitro assembly of synthetic chromosomes strongly increases with its size^19^. In addition, with size, the chance for type IIS restriction sites within the DNA of interest rises. In the 6 kb lac operon, already 4 natural BbsI and 3 Esp3I sites usually used in modular cloning systems are present. In the 151 kb region 37 BbsI, 8 BsaI and 37 Esp3I are present. Hence, at a certain size of the construct, either site removal^25^ or homology-based methods are required. Similar to modular cloning, assembly approaches based on yeast recombination suffer from laborious and time-consuming transfer back and forth between yeast and the organism of interest via in vitro methods^54^. With an iterative approach using modular assembly at the small scale to design customized parts and CRISPR SWAPnDROP for the assembly of the smaller parts to large chromosomes technical size limitations can be overcome and costs could be reduced drastically. With the transfer of 151 kb of DNA at an efficiency above 40%, we have shown that CRISPR SWAPnDROP is not limited in size within the limits of species-specific chromosome plasticity. In addition, the presented transfer of the *E. coli* lac operon into *V. natriegens* as well as the transfer of the GanB ORF from *D. dadantii* to *E. coli* is an example for a successful gain of function genome edit. Hence, it is possible to introduce desired properties from different organisms or a DNA library to construct tailor-made organisms.

## Methods

### Strains, media and reagents

*Escherichia coli* MG1655 was used for all genome editing experiments in *E. coli*, while *E. coli* DH5*α* was used for cloning purposes. For conjugation experiments, a precursor strain of MFD*pir* harbouring a stable RP4 conjugation machinery and additionally modified with *dapA* and *endA* knockouts was utilized^55^. All *E. coli* strains were cultivated in LB (10 g/L tryptone, 5 g/L yeast extract, 10 g/L NaCl) at 37 ^∘^C under aerobic conditions in flasks with shaking at 200 rpm. If needed, LB was supplemented with anhydrotetracycline (100 ng/ml), arabinose (1%) or 300 μM DAP. For experiments requiring minimal medium, M9 medium (33.7 mM Na_2_HPO_4_; 22 mM KH_2_PO_4_; 8.6 mM NaCl; 9.4 mM NH_4_Cl; 1 mM MgSO_4_; 0.3 mM CaCl_2_) supplemented with different carbon sources (lactose, arabinose, xylose, glucose: 0.5%) was used. For cloning, Q5 HF Polymerase (NEB), BsaI-HFv2 (NEB) and BbsI (NEB) were used. In this study, experiments with *V. natriegens* were carried out in *V. natriegens* ATCC 14048 ΔVNP12^56^. *V. natriegens* was cultivated in LB supplemented with V2 salts (204 mM NaCl, 4.2 mM KCl, 20.14 mM MgCl_2_) (LBv2) at 37 ^∘^C under aerobic conditions in flasks with shaking at 200 rpm. For growth of *V. natriegens* in minimal medium, M9 medium supplemented with different carbon sources (0.4%) and additional 2% NaCl was used. Experiments in *D. dadantii* were carried out in *D. dadantii* 3937. Cultivation was done in LB medium at 30 ^∘^C under aerobic conditions in flasks with shaking at 200 rpm. Preparation of chemically competent cells and transformation was carried out according to Green and Rogers^57^ and Stukenberg et al.^22^ for *E. coli* and *V. natriegens*, respectively. For *D. dadantii*, cr3Dd and pDrop were transferred via electroporation^58^. The pSwap plasmid was transferred via conjugation^59^.

### Sequencing of bacterial genomes and data analysis

Isolation of bacterial genomic DNA was performed via phenol/chloroform extraction according to Bruhn et al.^60^. Next-generation sequencing (NGS) was done by Eurofins Genomics Germany GmbH (Ebersberg, Germany). Reads were mapped and coverage was determined by the R package QuasR using a custom R script. The mutant reference genomes used for mapping were made using SnapGene on the basis of the *E. coli* MG1655 genome.

### pSwap assembly

The pSwap plasmid consists of seven modules each harbouring a part for the Swap and Drop recombination system: T1, T2 (sgRNA expression cassettes); H1*α*, H2*β*, HA, HB (homologous regions) and INS (insert fragment). For the T1 and T2 construction, 1 μl of complementary oligonucleotides (10 μM) consisting of the 20 bp target sequence and corresponding overhangs (see Supplementary Table 1), 10 μl of 10X T4-Ligase buffer (NEB) and 88 μl MilliQ water were incubated at 95^ ∘^C for 10 min and then slowly cooled down to room temperature. 1 μl of annealed oligonucleotides was used for cloning. For the construction of the other parts, PCR products using primer with corresponding overhangs were used (see Supplementary Table 1). Approximately 40 fmol (~25 ng/1000 bp) of PCR products were utilized for cloning. Annealed oligonucleotides or PCR products were mixed with 40 fmol of the corresponding entry vectors, 1X T4-Ligase buffer (NEB), 1 μl (20 units) BsaI-HFv2 (NEB), 1 μl (400 units) T4-Ligase (NEB) and MilliQ water to a final volume of 20 μl. The mix was incubated for 2–5 h at 37 ^∘^C for in vitro assembly. In case of internal BsaI sites in the inserts, a final 30 min ligation step at 16 ^∘^C prior to heat inactivation is added for optimal Ligase activity and reduced BsaI activity. The mix was transformed into chemically competent DH5*α* cells and subsequently plated on LB Agar supplemented with 1mM IPTG and 250 μM X-gal. White colonies were tested for a successful integration into the entry vectors via sanger sequencing. Antibiotic concentrations used for cultivation of each part is seen in Table Supplementary 1. If needed, H*α*- and H*β* homologies for H1*α* and H2*β* were added as an additional PCR fragment without fixed overhangs upstream of the 5′-end of the H1 homology or downstream of the 3′-end of the H2 homology. For the pSwap assembly, 40 fmol of each part (T1, T2, H1*α*, H2*β*, HA, HB, INS), 1X T4-Ligase buffer, 1 μl (10 units) BbsI (NEB), 1 μl (400 units) T4-Ligase and MilliQ water were incubated in a final volume of 20 μl for 5–7 h at 37 ^∘^C. In case of internal BbsI sites in the inserts, a final 30 min ligation step at 16 ^∘^C prior to heat inactivation can be added for optimal Ligase activity and reduced BbsI activity. The restriction-ligation could then be transformed into a cloning (DH5*α*) or directly into the desired wild-type strain. Successfully assembled pSwap plasmids were verified by purple colonies after transformation. Different parts of the deoxyviolacein pathway are distributed on different modules. However, only when the modules are correctly assembled into the pSwap, a functional deoxyviolacein pathway result in purple colonies. Antibiotics were used depending on the corresponding H2*β* (see Supplementary Table 1). For insertion or deletion experiments, T2 was not essential, but still mandatory for the pSwap assembly. Therefore, a random DNA sequence, which was not present in the strains’ chromosomal sequence, was cloned into the entry vector and used for the assembly. Homologies (H1, H2) for the loading of the pSwap were also not needed, but a recombination between those random sequences was important to maintain the integrity of the pSwap plasmid during genome modifications. Therefore, identical 175 bp random DNA sequences were cloned into H1*α* and H2*β* (HR, see Fig. 1a). In general homology arms of 500^48^ and 1500 bp^27^ were used for *E. coli* and *V. natriegens*, respectively. As all parts were digested with BsaI and BbsI for cloning, the presence of those restriction sites within the parts (e.g. the homologies or insert fragment) were avoided. Therefore, in some cases the size for homology arms were chosen between 50 and 500 bp for *E. coli* and between 1000 and 1500 bp for *V. natriegens* to avoid present restriction sites. A list with Primers, which were used for the construction of each individual pSwap plasmid can be found in the supplementary data.

### CRISPR SWAPnDROP genome editing protocol (Indel/Swap)

*E. coli* MG1655 was first transformed with the helper plasmid cr3Ec. For all CRISPR/Cas9 experiments, cr3Ec harbouring the P_*tet*_ repressor (TetR) needed to be transformed first to establish P_*tet*_ repression prior to the transformation with pSwap. Double transformation with pSwap and cr3Ec lead to dysfunctional components in the Cas9 system^14^. After transformation with cr3Ec, *E. coli* MG1655 cr3Ec was transformed either directly with the pSwap restriction-ligation mix or the prepared pSwap plasmid. The transformants were grown on LB Agar with appropriate antibiotics (see Supplementary Table 2) at 37 ^∘^C overnight. The plates were then incubated for 2–5 h at room temperature allowing the colonies to form a purple colour. Subsequently, purple colonies were inoculated in LB with 100 ng/ml anhydrotetracyclin (aTet), 1% arabinose and appropriate antibiotics (see Supplementary Table 2). Cultures were grown at 37 ^∘^C shaking (200 rpm) overnight and plated on LB Agar with the same antibiotics at 37 ^∘^C. After ~16 h, the plates were incubated at room temperature for 2–5 h now allowing the colonies to form a green colour. Green colonies were then tested for a successful editing event via PCR and Sanger sequencing.

### *V*ibrio natriegens genome editing protocol (Indel/Swap)

The *V. natriegens* strain ATCC 14048 ΔVNP12 was first transformed with cr3Vn. Subsequently, this strain was transformed with pSwap Cm^*R*^ and incubated on LBv2 Agar with corresponding antibiotics at 37 ^∘^C overnight (see Supplementary Table 3). The plates were incubated at room temperature for several hours to develop its purple colour. Purple colonies were inoculated and grown until OD_600_ reached 4 or overnight in LBv2 with appropriate antibiotics at 37 ^∘^C and 200 rpm shaking. Induction of the *λ*RED genes was carried out by adding 0.4% arabinose for 1–2 h followed by the induction of the Cas9 gene and the sgRNA with aTet (80 ng/ml) for further 2–4 h at 37 ^∘^C. Subsequently, the cultures were plated on LBv2 with 80 ng/ml aTet, 0.4% arabinose, antibiotics (see Supplementary Table 3) and grown at 37 ^∘^C overnight. The following day, colonies were incubated at room temperature for several hours or until the next day. Green colonies were then tested via PCR and Sanger sequencing.

### Dickeya dadantii genome editing protocol (Indel/Swap)

*D. dadantii* 3937 was first transformed with cr3Dd via electrotransformation. After transformation/conjugation of the pSwap, cells were incubated on LB Agar with corresponding antibiotics at 30 ^∘^C overnight (see Supplementary Table 4). Purple colonies were inoculated in LB medium with appropriate antibiotics and grown overnight at 30 ^∘^C. Induction of the *λ*RED genes was carried out by adding 0.4% arabinose for 1–2 h followed by the induction of the Cas9 gene and the sgRNA with aTet (80 ng/ml) for further 2–4 h at 30 ^∘^C. The culture was then plated on LB Agar supplemented with 100 ng/ml aTet, 0.4% arabinose and antibiotics (see Supplementary Table 4).

### Conjugation and drop protocol

For the transfer and integration of chromosomal DNA, the loading of the pSwap plasmid with a chromosomal region was carried out in the donor strain *E. coli* MFD*pir* Δ *dapA* Δ *endA*, harbouring the RP4 conjugation system^55^. Simultaneously, the acceptor strain was transformed with the helper plasmids cr3Ec and pDrop. Similar to the sgRNA level 1 parts (T1, T2) of the pSwap assembly, the spacer sequence (T3) for the counter-selection in the acceptor strain needed to be cloned into the pDrop entry vector and subsequently verified by sanger sequencing. Both, the donor strain harbouring the loaded pSwap plasmid (pSwap’) and the RP4 conjugation system as well as the acceptor strain harbouring the helper plasmids were cultivated in LB with correct antibiotics at 37 ^∘^C overnight (see Supplementary Table 2). The culture of the *E. coli* donor strain was additionally supplemented with 300 μM diaminopimelic acid (DAP). The donor and acceptor strains were diluted to an OD_600_ of 3 with fresh LB medium after washing and then mixed with a 1:1 ratio. Subsequently, the mix was spotted on LB Agar plates only supplemented with 300 μM DAP and incubated at 37^ ∘^C overnight. The spot was scrapped off the plate and washed several times with LB medium to remove residual DAP. Selection for successful conjugation was performed on LB Agar with appropriate antibiotics (see Supplementary Table 2). After the conjugation of the pSwap’ plasmid, the acceptor strain was inoculated and directly induced in LB with the corresponding antibiotics for the selection of cr3Ec and pDrop (see Supplementary Table 2). Selection for the pSwap was omitted, as during the excision of the recombination template, the pSwap plasmid was destroyed. Correct function of the CRISPR/Cas9- and *λ*RED-systems was ensured by the cut and reconstitution of the pDrop non-functional mScarlet gene yielding red colonies. Induction was again carried out with 1% arabinose and 100 ng/ml aTet. The induced acceptor strain was cultivated overnight at 37 ^∘^C and then plated on LB with the same antibiotics. Red colonies were tested for the integration into chromosome via PCR and sanger sequencing. Conjugation and subsequent chromosome integration from *E. coli* to *V. natriegens* were carried out similarly. For *V. natriegens* pDropVn containing a gentamycin resistance cassette, as selection with ampicillin did not provide satisfying results. For the conjugation, the stationary phase cultures diluted to an OD_600_ of 3 were mixed with a 1:9 ratio (*V. natriegens*:*E. coli*) and then spotted on LBv2 agar plates, also supplemented with DAP. *V. natriegens* now harbouring the pSwap’ plasmid, cr3Vn and pDropVn were inoculated and grown overnight at 37 ^∘^C with corresponding antibiotics (see Supplementary Table 3). The next day, induction was performed in the stationary phase culture by inducing first the *λ*RED recombination with 0.4% arabinose for 1–2 h and then the CRISPR/Cas9 with 80 ng/ml aTet for another 2–4 h. The induced culture was then plated on LBv2 agar with antibiotics as well as 0.4% arabinose and 80 ng/ml aTet. Red colonies were tested via PCR and Sanger sequencing. Conjugational transfer of pSwap from *V. natriegensrecJ*::RP4 to *E. coli* was performed in analogy to the reverse direction. For conjugation spots, LB instead of LBv2 agar plates were used. Conjugation and subsequent chromosome integration from *E. coli* to *D. dadantii* were carried out in analogy to the *V. natriegens* protocol, but using LB with *D. dadantii* specific antibiotic concentrations (see Supplementary Table 4) and 30 ^∘^C for optimal growth. Due to the lower growth rate of *D. dadantii* compared to *V. natriegens*, a 1:1 mix ratio was used during conjugation.

### Reporting summary

Further information on research design is available in the Nature Research Reporting Summary linked to this article.

## Supplementary information

Supplementary Information

Reporting Summary

Description of Additional Supplementary Files

Supplementary Data 1

## Data Availability

The DNA sequencing data for edit verification generated in this study have been deposited in the sequence read archive (SRA) database under accession code PRJNA824474. The plot raw data generated in this study are provided in the Supplementary Information/Source data file. Plasmid maps and cloning templates for pSwap construction can be found in the supplementary data. Source data are provided with this paper.

## Source data

Source Data
