# Supplementary material for: A multifunctional system for genome editing and large-scale interspecies gene transfer
Source: Nat Commun. 2022 Jun 14;13:3430. doi: 10.1038/s41467-022-30843-1 (PMC9198041; doi:10.1038/s41467-022-30843-1)
Supplement: Supplementary file 1 — Supplementary Information [file 41467_2022_30843_MOESM1_ESM.pdf]

CRISPR SWAPnDROP - A multifunctional system for genome editing and large-scale interspecies gene transfer

Marc Teufel[1], Carlo A. Klein[1], Maurice Mager[1], and Patrick Sobetzko[1,*]

[1]Philipps Universität Marburg, Synthetic Microbiology Center Marburg (SYNMIKRO), Marburg, 35043, Germany
[*]patrick.sobetzko@synmikro.uni-marburg.de

ABSTRACT

CRISPR SWAPnDROP extends the limits of genome editing to large-scale in-vivo DNA transfer between bacterial species. Its modular platform approach facilitates species specific adaptation to confer genome editing in various species. In this study, we show the implementation of the CRISPR SWAPnDROP concept for the model organism *Escherichia coli*, the fast growing *Vibrio natriegens* and the plant pathogen *Dickeya dadantii*. We demonstrate the excision, transfer and integration of large chromosomal regions between *E. coli*, *V. natriegens* and *D. dadantii* without size-limiting intermediate DNA extraction. CRISPR SWAPnDROP also provides common genome editing approaches comprising scarless, marker-free, iterative and parallel insertions and deletions. The modular character facilitates DNA library applications, and recycling of standardized parts. Its multi-color scarless co-selection system significantly improves editing efficiency and provides visual quality controls throughout the assembly and editing process.

Supplementary Table 1. Primer overhangs and antibiotics used for pSwap parts construction

Part	FW Primer overhang (5'-overhang)	REV Primer overhang (5'-overhang)	Antibiotic (µg/ml)
T1	GAggtctcA**GCAC**	GAggtctcA**AAAC**	kanamycin (35)
T2	GAggtctcA**GCAC**	GAggtctcA**AAAC**	kanamycin(35)
H1α	GAggtctcA**GGAG**-<u>GCTCGATCGCTAATCGTTATCGG</u>	GAggtctcA**ACGG**	kanamycin(35)
H2β	GAggtctcA**GGAG**	GAggtctcA**AGCG**-<u>GCTCGATCGCTAATCGTTATCGG</u>	chloramph.(18) gentamycin (10)
HA	GAggtctcA**CCGT**	GAggtctcA**CTCC**	kanamycin(35)
HB	GAggtctcA**CCGT**	GAggtctcA**AGCG**	kanamycin(35)
INS	GAggtctcA**GGAG**	GAggtctcA**AGCG**	kanamycin(35)
HAIB	GAggtctcA**CCGT**	GAggtctcA**AGCG**	kanamycin(35)

Lower-case and red letters indicate the BsaI restriction site and the required overhangs, respectively. Underlined letters represent the Cas9 cut site (TD) and have to be added to the primer if homology α and β are used.

Supplementary Table 2. Plasmids and corresponding antibiotic resistance for *E. coli*

Plasmid	Antibiotic resistance (µg/ml)	Additional information (Inducer)
cr3Ec	Kanamycin (35)	aTet/Arabinose (Cas9/λRED)
pSwap Cm^R	Chloramphenicol (18)	aTet (sgRNA)
pSwap Gm^R	Gentamycin (10)	aTet (sgRNA)
pDrop	Ampicillin (100)	aTet (sgRNA)

Supplementary Table 3. Plasmids and corresponding antibiotic resistance for *V. natriegens*

Plasmid	Antibiotic resistance (µg/ml)	Additional information (Inducer)
cr3Vn	Kanamycin (200)	aTet/Arabinose (Cas9/λRED)
pSwap Cm^R	Chloramphenicol (2)	aTet (sgRNA)
pDropVn	Gentamycin (50)	aTet (sgRNA)

Supplementary Table 4. Plasmids and corresponding antibiotic resistance for *D. dadantii*

Plasmid	Antibiotic resistance (µg/ml)	Additional information (Inducer)
cr3Dd	Kanamycin (7)	aTet/Arabinose (Cas9/λRED)
pSwap Cm^R	Chloramphenicol (7)	aTet (sgRNA)
pDrop	Ampicillin (20)	aTet (sgRNA)

Supplementary Table 5. Workflow of CRISPR SWAPnDROP. The numbers in brackets are the days using a present DNA library. Gene editing and gene transfer share the same protocol until day 7(4) (Black line). The workflow for the gene transfer continues at day 8(5).

Day	Expenditure of time	Task
1	2 h	PCR (homologies, insert and targets), cloning, transformation in cloning strain
2	1 h	Colony screening and over-night culture
3	1 h	Plasmid preparation and sequencing
4(1)	1 h	Assembly of pSwap and transformation in destination strain
5(2)	5 min	Purple colony induction in liquid medium
6(3)	5 min	Plating of over-night culture
7(4)	1 h	PCR screening of green colonies and sequencing
8(5)	1 h	Conjugation into the acceptor strain and plating
9(6)	5 min	Green colony induction in liquid medium
10(7)	5 min	Plating of over-night culture
11(8)	1 h	PCR screening of red colonies and sequencing

Supplementary Table 6. Comparison of Cas9 RED based systems and CRISPR SWAPnDROP regarding suppressor detection. For the 'system state' column (+) and (-) indicates the presence or absence of successful editing events, respectively. For the other columns, (+) and (-) indicates the positive or negative expectation for observed colonies.

System state	Cas9 RED	CRISPR SWAPnDROP
fully functional (+)	white colonies (+)	green colonies (+)
Cas9 defect (-)	white colonies (+)	violet colonies (-)
RED defect (-)	no colonies	no colonies
deletion in plasmid (-)	white colonies (+)	white colonies (-)

Supplementary Table 7. Feature comparison of current systems. A plus symbol indicates the capability of the system. A plus in brackets indicates a conditional capability e.g. in combination with other subsequent methods. A minus sign indicates the lack of the feature.

Feature	SWAPnDROP	REXER	RED	CRMAGE	No-Scar
scarless	+	+	-	+	+
marker-free	+	-	-	+	+
indels	+	+	+	+	+
large fragments	+	+	-	-	-
rearrangements	+	-	-	-	-
DNA transfer	+	-	-	-	-
modular	+	-	-	-	-
iterative	+	+	(+)	+	+
multiplex	+	-	-	+	-

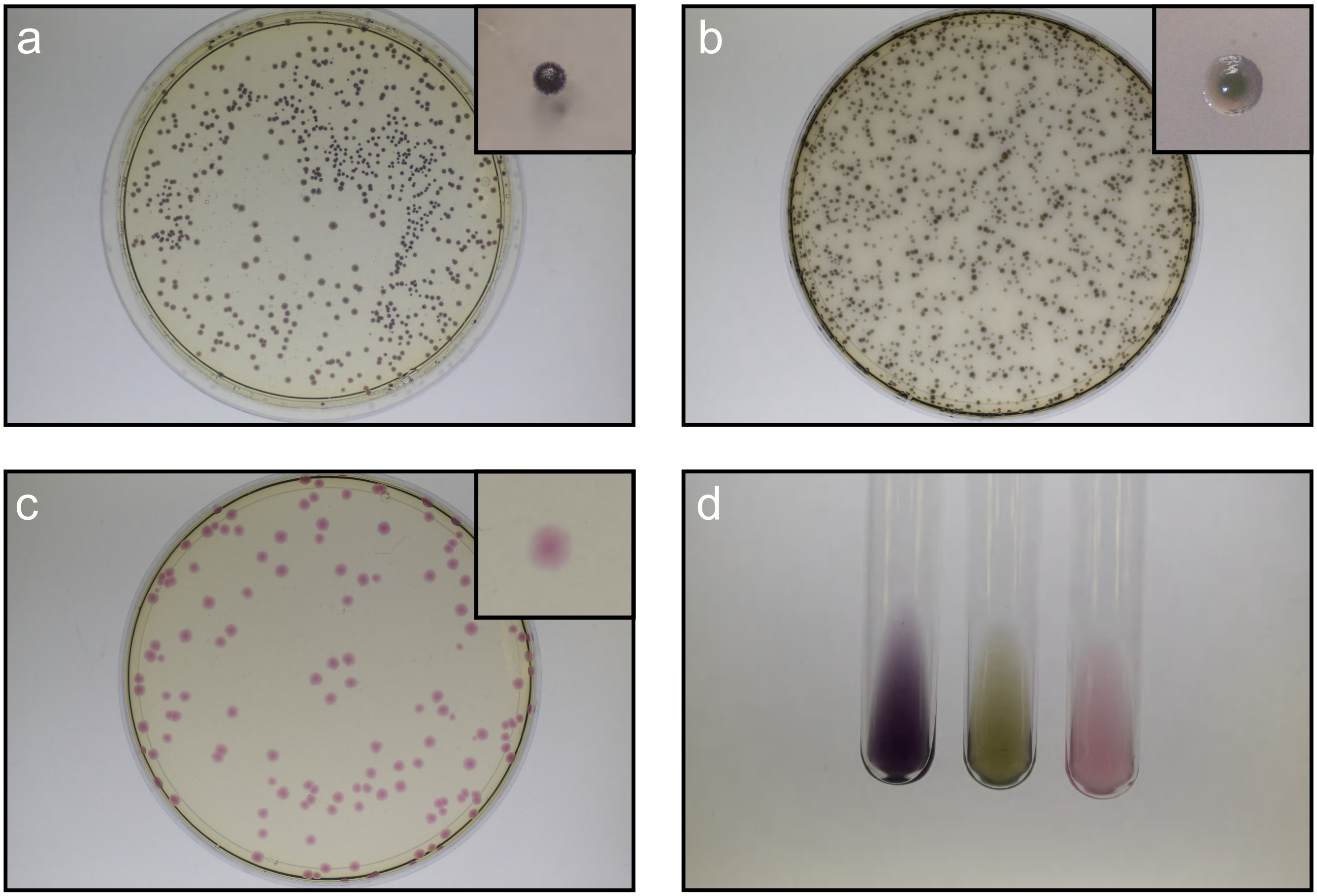

Supplementary Figure 1. Colony and culture colors of the multi-color selection system. a Shown is a picture of purple colonies representing a successful pSwap assembly. **b** Shown is a picture of green colonies after a successful editing event (SWAP). **c** Shown is a picture of red colonies after a successful editing event (DROP). **d** Shown is a picture of bacterial cultures, each with one of the 3 different colors (purple, green, red) used for selection.

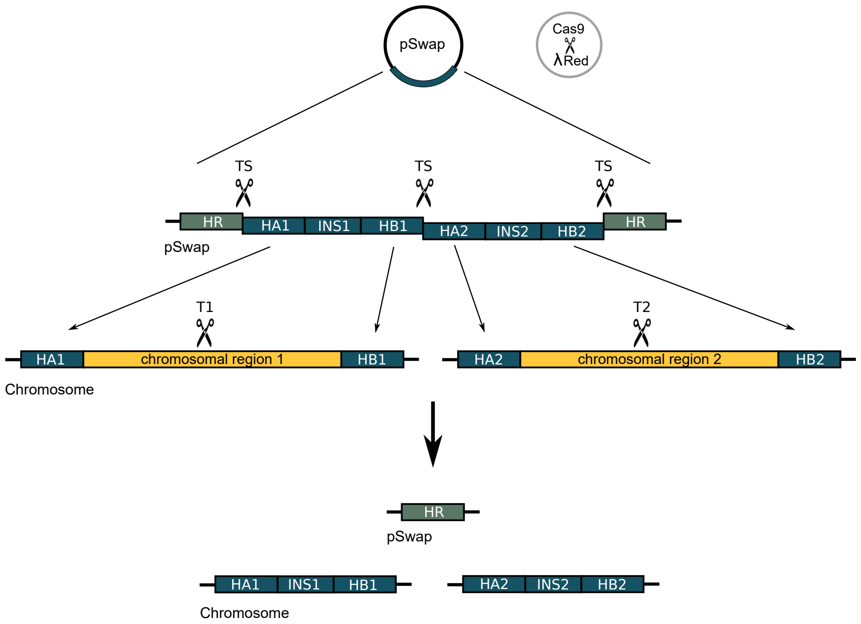

Supplementary Figure 2. Mechanism of CRISPR SWAPnDROP recombination for multiplex genome editing. CRISPR SWAPnDROP can also be used for the simultaneous edit of two chromosomal regions. For this purpose, two templates (HA-INS-HB) for recombination are assembled together and separated by an additional Cas9 excision site between HB1 and HA2. T1 and T2 sgRNAs targeting two different chromosomal loci are used for counter-selection.

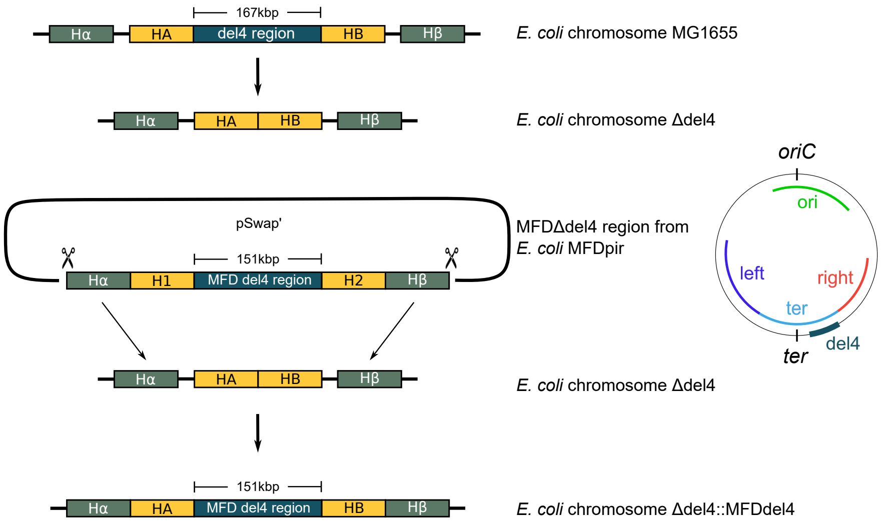

Supplementary Figure 3. Transfer and integration of a 151kb chromosomal region via CRISPR SWAPnDROP. To demonstrate the transfer of large chromosomal regions, an *E. coli* MG1655 strain was first generated lacking a 167kb chromosomal region (del4) near the terminus of the *E. coli* chromosome (*E. coli* MG1655 Δdel4). *E. coli* MFD*pir* was then used to load the pSwap plasmid with the MFDdel4 region (151kb) using H1 and H2, which are equivalent to HA and HB in the *E. coli* MG1655 Δdel4 strain. The loaded pSwap' was then conjugated into *E. coli* MG1655 Δdel4. For the reconstitution of the *E. coli* MG1655 Δdel4 strain, Hα and Hβ were used to drop the 151kb region into the deleted del4 region. This resulted in the strain *E. coli* MG1655 Δdel4::MFDdel4, which lacks small regions between Hα/HA and HB/Hβ.

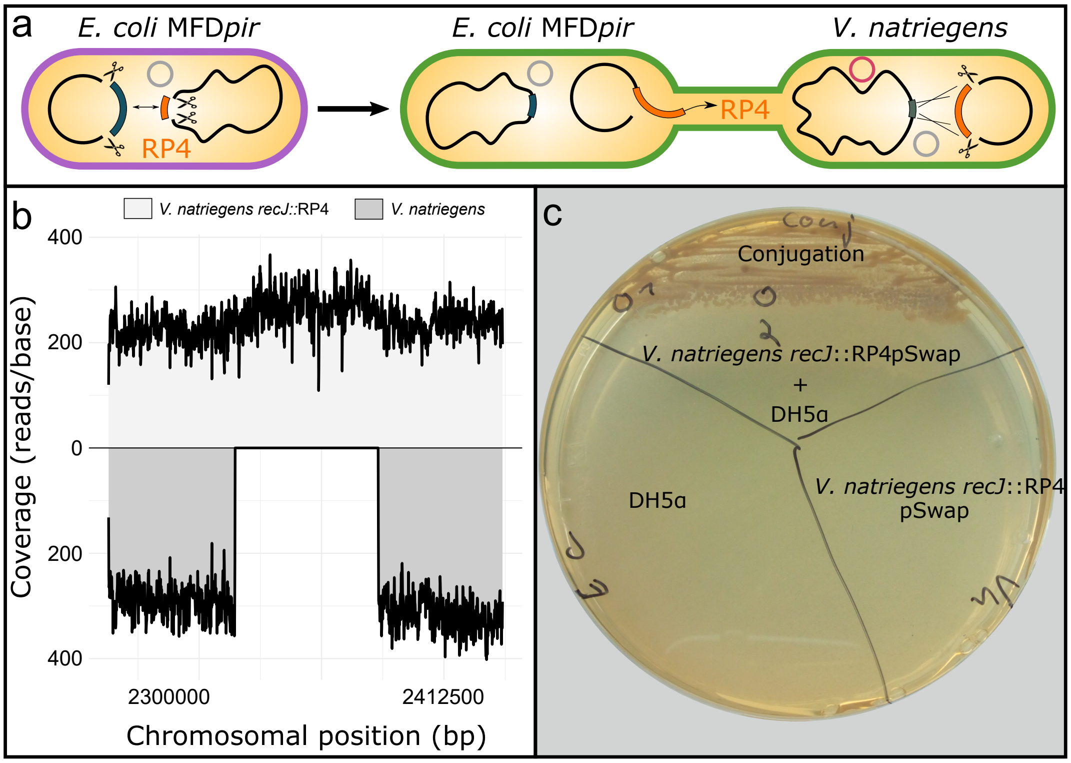

Supplementary Figure 4. **Transfer of the RP4 conjugation system to *V. natriegens*** **a** Scheme of the swap and conjugational transfer of the RP4 conjugation system from *E. coli*MFDpir to *V. natriegens* and subsequent integration.. **b** Shown is the next-generation sequencing (NGS) coverage (reads/base) of *V. natriegens recJ*::RP4 (light grey) and *V. natriegens* (dark grey) at the RP4 insert locus (*recJ*) mapped against the *V. natriegens recJ*::RP4 reference genome. **c** Conjugation test of pSwap from *V. natriegens recJ*::RP4 to *E. coli* DH5α. Controls for donor and acceptor strains are indicated.
