# Supplementary material for: A multifunctional system for genome editing and large-scale interspecies gene transfer
Source: Nat Commun. 2022 Jun 14;13:3430. doi: 10.1038/s41467-022-30843-1 (PMC9198041; doi:10.1038/s41467-022-30843-1)
Supplement: Supplementary file 2 — Reporting Summary [file 41467_2022_30843_MOESM2_ESM.pdf]

nature portfolio

Corresponding author(s): NCOMMS-21-23048B-Z

Last updated by author(s): 10-05-2022

Reporting Summary

Nature Portfolio wishes to improve the reproducibility of the work that we publish. This form provides structure for consistency and transparency in reporting. For further information on Nature Portfolio policies, see our Editorial Policies and the Editorial Policy Checklist.

Statistics

For all statistical analyses, confirm that the following items are present in the figure legend, table legend, main text, or Methods section.

n/a	Confirmed	
☐	☒	The exact sample size (*n*) for each experimental group/condition, given as a discrete number and unit of measurement
☐	☒	A statement on whether measurements were taken from distinct samples or whether the same sample was measured repeatedly
☒	☐	The statistical test(s) used AND whether they are one- or two-sided<br>*Only common tests should be described solely by name; describe more complex techniques in the Methods section.*
☒	☐	A description of all covariates tested
☒	☐	A description of any assumptions or corrections, such as tests of normality and adjustment for multiple comparisons
☐	☒	A full description of the statistical parameters including central tendency (e.g. means) or other basic estimates (e.g. regression coefficient) AND variation (e.g. standard deviation) or associated estimates of uncertainty (e.g. confidence intervals)
☒	☐	For null hypothesis testing, the test statistic (e.g. *F*, *t*, *r*) with confidence intervals, effect sizes, degrees of freedom and *P* value noted<br>*Give P values as exact values whenever suitable.*
☒	☐	For Bayesian analysis, information on the choice of priors and Markov chain Monte Carlo settings
☒	☐	For hierarchical and complex designs, identification of the appropriate level for tests and full reporting of outcomes
☒	☐	Estimates of effect sizes (e.g. Cohen's *d*, Pearson's *r*), indicating how they were calculated

Our web collection on statistics for biologists contains articles on many of the points above.

Software and code

Policy information about availability of computer code

Data collection	sequence and annotation files of bacterial genomes were taken from NCBI bacterial sequence database.
Data analysis	Custom R scripts (R 3.2.2) were used for data analysis. The QuasR R package (v1.6.2) was used for sequence read mapping. The R code only includes plotting and standard DNA mapping function and do not harbor any algorithms relevant for publishing. For planning of plasmids SnapGene (v1.1.3) was used.

For manuscripts utilizing custom algorithms or software that are central to the research but not yet described in published literature, software must be made available to editors and reviewers. We strongly encourage code deposition in a community repository (e.g. GitHub). See the Nature Portfolio guidelines for submitting code & software for further information.

Data

Policy information about availability of data

All manuscripts must include a data availability statement. This statement should provide the following information, where applicable:

- Accession codes, unique identifiers, or web links for publicly available datasets
- A description of any restrictions on data availability
- For clinical datasets or third party data, please ensure that the statement adheres to our policy

data, including chromosomal regions, cloning templates and plasmid maps used in this study are provided in supplementary data.

Field-specific reporting

Please select the one below that is the best fit for your research. If you are not sure, read the appropriate sections before making your selection.

☒ Life sciences ☐ Behavioural & social sciences ☐ Ecological, evolutionary & environmental sciences

For a reference copy of the document with all sections, see nature.com/documents/nr-reporting-summary-flat.pdf

Life sciences study design

All studies must disclose on these points even when the disclosure is negative.

Sample size	For all genome edits three biological replicates were perfomed. Three replicates are sufficient to support the practical reliability of genome editing. For editing efficiency, positive and negative edits were determined by clear phenotypic difference in colonies (blue and white). Colony numbers are not predictable. Therefore, sample size differs between samples. However, the large sample size allows for precise estimations of efficiencies.
Data exclusions	no data was excluded.
Replication	For all genome edits three biological replicates were performed. The nature of the experiments does not allow any statement about reproducibility, as the variance of the method is part of the study and is described in the text and methods sections.
Randomization	not applicable for this study. In the study, bacterial populations were investigated which are by definition randomized.
Blinding	In this study bacteria are investigated. Hence, no blinding is necessary, The study was performed by several people crosschecking results.

Reporting for specific materials, systems and methods

We require information from authors about some types of materials, experimental systems and methods used in many studies. Here, indicate whether each material, system or method listed is relevant to your study. If you are not sure if a list item applies to your research, read the appropriate section before selecting a response.

Materials & experimental systems

n/a	Involved in the study	
☒	☐	Antibodies
☒	☐	Eukaryotic cell lines
☒	☐	Palaeontology and archaeology
☒	☐	Animals and other organisms
☒	☐	Human research participants
☒	☐	Clinical data
☒	☐	Dual use research of concern

Methods

n/a	Involved in the study	
☒	☐	ChIP-seq
☒	☐	Flow cytometry
☒	☐	MRI-based neuroimaging
