# Supplementary material for: A multifunctional system for genome editing and large-scale interspecies gene transfer
Source: Nat Commun. 2022 Jun 14;13:3430. doi: 10.1038/s41467-022-30843-1 (PMC9198041; doi:10.1038/s41467-022-30843-1)
Supplement: Supplementary file 3 — Description of Additional Supplementary Files [file 41467_2022_30843_MOESM3_ESM.pdf]

Title: Supplementary Data 1

Description: Primers used in this study
