# Supplementary material for: A multifunctional system for genome editing and large-scale interspecies gene transfer
Source: Nat Commun. 2022 Jun 14;13:3430. doi: 10.1038/s41467-022-30843-1 (PMC9198041; doi:10.1038/s41467-022-30843-1)
Supplement: Supplementary file 5 — Source Data [file 41467_2022_30843_MOESM5_ESM.zip › Templates.zip!/Templates/homology_1_alpha_template.dna.pdf]

Sequence: homology_1_alpha_template.dna (Linear / 164 bp)
Enzymes: MoClo (6 of 646 total)
Features: 11 visible, 12 total

Unique Cutters **Bold**

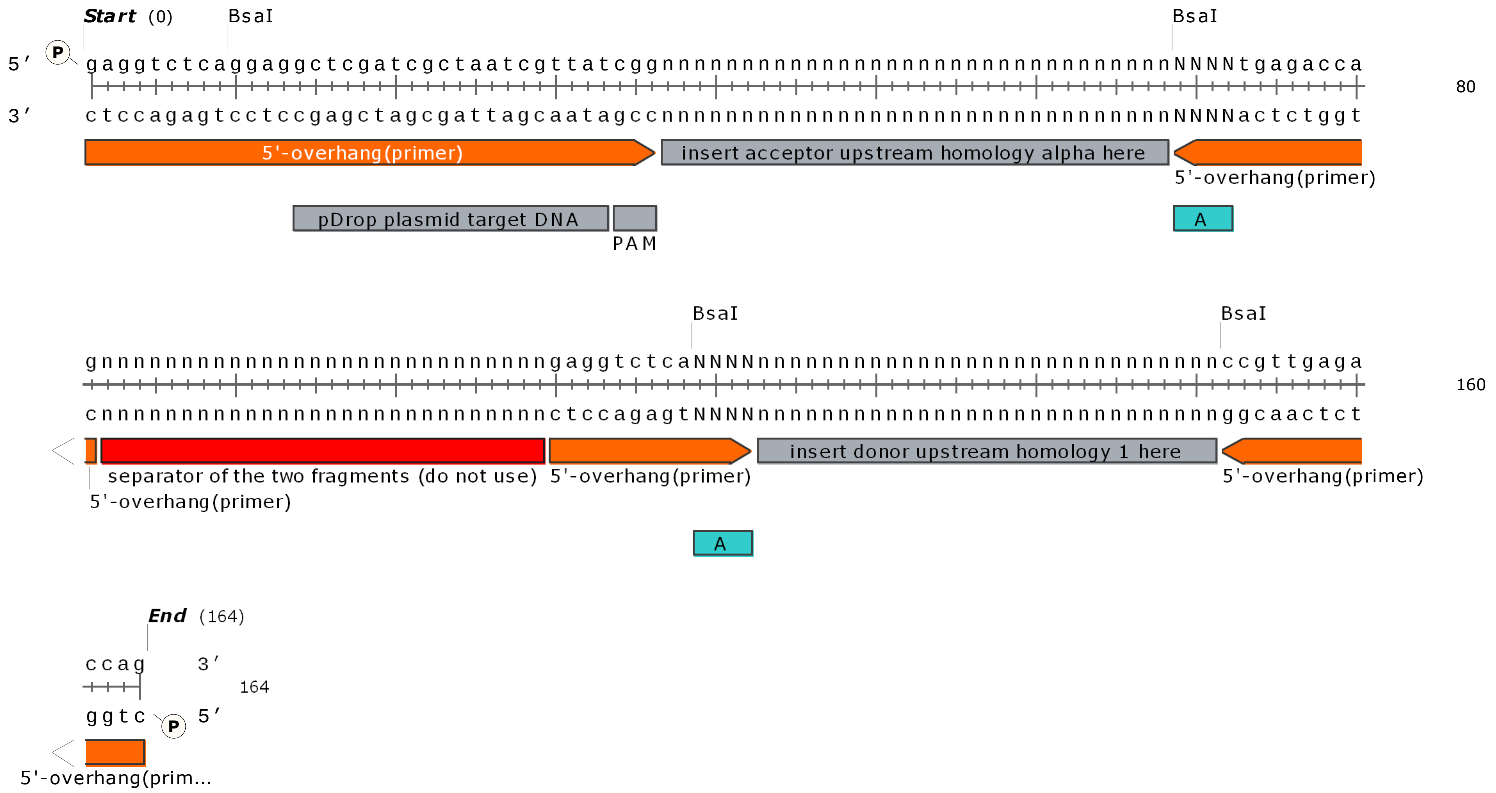

	Feature	Location	Size (bp)	Type
	**source**	1 .. 164	164	source
	/organism = synthetic DNA construct			
	/mol_type = other DNA			
✓	**5'-overhang(primer)**	1 .. 36	36	misc_feature
✓	**pDrop plasmid target DNA**	14 .. 33	20	misc_feature
✓	**PAM**	34 .. 36	3	misc_feature
✓	**insert acceptor upstream homology alpha here**	37 .. 68	32	misc_feature
✓	**5'-overhang(primer)**	69 .. 81	13	misc_feature
✓	**A**	69 .. 72	4	misc_feature
✓	**separator of the two fragments (do not use)**	82 .. 109	28	misc_feature
✓	**5'-overhang(primer)**	110 .. 122	13	misc_feature
✓	**A**	119 .. 122	4	misc_feature
✓	**insert donor upstream homology 1 here**	123 .. 151	29	misc_feature
✓	**5'-overhang(primer)**	152 .. 164	13	misc_feature
