# Supplementary material for: A multifunctional system for genome editing and large-scale interspecies gene transfer
Source: Nat Commun. 2022 Jun 14;13:3430. doi: 10.1038/s41467-022-30843-1 (PMC9198041; doi:10.1038/s41467-022-30843-1)
Supplement: Supplementary file 5 — Source Data [file 41467_2022_30843_MOESM5_ESM.zip › Templates.zip!/Templates/homology_1_template.dna.pdf]

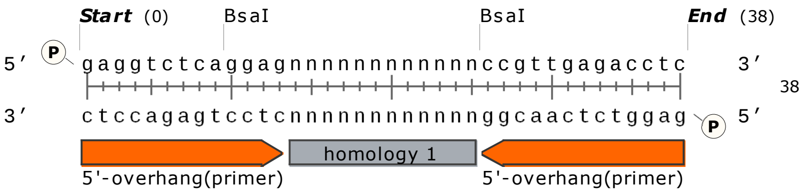
Start (0)
BsaI
BsaI
End (38)
5′
P
gaggtctcaggagnnnnnnnnnnnnccgttgagacctc
3′
38
3′
ctccagagtcctcnnnnnnnnnnnnggcaactctggag
P
5′
homology 1
5'-overhang(primer)
5'-overhang(primer)

	Feature	Location	Size (bp)	Color	Direction	Type
	**source**	1 .. 38	38		⟷	source
	/organism = unspecified					
	/mol_type = genomic DNA					
✓	**5'-overhang(primer)**	1 .. 13	13		→	misc_feature
✓	**homology 1**	14 .. 25	12		⟷	misc_feature
✓	**5'-overhang(primer)**	26 .. 38	13		←	misc_feature
	**source**	26 .. 26	1		⟷	source
	/organism = pKDsgRNA-ack					
	/mol_type = other DNA					
	**source**	26 .. 26	1		⟷	source
	/organism = pTKIP-cat					
	/mol_type = other DNA					
