# Supplementary material for: A multifunctional system for genome editing and large-scale interspecies gene transfer
Source: Nat Commun. 2022 Jun 14;13:3430. doi: 10.1038/s41467-022-30843-1 (PMC9198041; doi:10.1038/s41467-022-30843-1)
Supplement: Supplementary file 5 — Source Data [file 41467_2022_30843_MOESM5_ESM.zip › Templates.zip!/Templates/homology_2_beta_template.dna.pdf]

Sequence: homology_2_beta_template.dna (Linear / 168 bp)
Enzymes: MoClo (6 of 646 total)
Features: 11 visible, 12 total

Unique Cutters **Bold**

Start (0) BsaI BsaI

5' gaggtctcaggagnnnnnnnnnnnnnnnnnnnnnnnnnnnnnNNNNtgagaccagnnnnnnnnnnnnnnnnnnnnnnnnn 80

3' ctccagagtcctcnnnnnnnnnnnnnnnnnnnnnnnnnnnnnNNNNactctggtcnnnnnnnnnnnnnnnnnnnnnnnnn

5'-overhang(primer) | insert donor homology 2 here | 5'-overhang(primer) | separator for the two fragments (do not use)

A

BsaI BsaI

nnnnnnnnnngaggtctcaNNNNnnnnnnnnnnnnnnnnnnnnnnnnnnnnnccgataacgattagcgatcgagccgctt 160

nnnnnnnnnnctccagagtNNNNnnnnnnnnnnnnnnnnnnnnnnnnnnnnnggctattgctaatcgctagctcggcgaa

separator fo... | 5'-overhang(primer) | insert acceptor homology beta here | 5'-overhang(primer)

A | PAM | pDrop plasmid target DNA

End (168)

gagaccag 3' 168

ctctggtc 5'

5'-overhang(primer)

	Feature	Location	Size (bp)			Type
	**source**	1 .. 168	168	white	⟷	source
	/organism = synthetic DNA construct					
	/mol_type = other DNA					
✓	**5'-overhang(primer)**	1 .. 13	13	orange	→	misc_feature
✓	**insert donor homology 2 here**	14 .. 42	29	grey	⟷	misc_feature
✓	**5'-overhang(primer)**	43 .. 55	13	orange	←	misc_feature
✓	**A**	43 .. 46	4	cyan	⟷	misc_feature
✓	**separator for the two fragments (do not use)**	56 .. 90	35	red	⟷	misc_feature
✓	**5'-overhang(primer)**	91 .. 103	13	orange	→	misc_feature
✓	**A**	100 .. 103	4	cyan	⟷	misc_feature
✓	**insert acceptor homology beta here**	104 .. 132	29	grey	⟷	misc_feature
✓	**5'-overhang(primer)**	133 .. 168	36	orange	←	misc_feature
✓	**PAM**	133 .. 135	3	grey	⟷	misc_feature
✓	**pDrop plasmid target DNA**	136 .. 155	20	grey	⟷	misc_feature
