# Supplementary material for: A multifunctional system for genome editing and large-scale interspecies gene transfer
Source: Nat Commun. 2022 Jun 14;13:3430. doi: 10.1038/s41467-022-30843-1 (PMC9198041; doi:10.1038/s41467-022-30843-1)
Supplement: Supplementary file 5 — Source Data [file 41467_2022_30843_MOESM5_ESM.zip › Templates.zip!/Templates/homology_AIB_dual_edit_template.dna.pdf]

Sequence: homology_AIB_dual_edit_template.dna (Linear / 414 bp)
Enzymes: MoClo (6 of 646 total)
Features: 33 visible, 48 total

Unique Cutters **Bold**

Start (0) BsaI BsaI BsaI

5′ gaggtctcaccgtnnnnnnnnnnnnnnnnnnnnnnnnnnNNNNtgagacctcnnnnnnnnnnnnnnnnngaggtctcaNN 80
3′ ctccagagtggcannnnnnnnnnnnnnnnnnnnnnnnnnnNNNNactctggagnnnnnnnnnnnnnnnnnctccagagtNN

5'-overhang(primer) | homology A (edit 1) | Separator(do not use) | 5'-overhang(primer)
5'-overhang(primer) | A | A

BsaI BsaI

NNnnnnnnnnnnnnnnnnnnnnnnnnnNNNNtgagacctcnnnnnnnnnnngaggtctcaNNNNnnnnnnnnnnnnnnnn 160
NNnnnnnnnnnnnnnnnnnnnnnnnnnNNNNactctggagnnnnnnnnnnnctccagagtNNNNnnnnnnnnnnnnnnnn

5'-overhang(primer) | insert (edit 1) | 5'-overhang(primer) | homology B (edit 1)
A | B | 5'-overhang(primer) | B

BsaI BsaI

nnnnnnnnnnnnnnNNNNtgagacctcnnnnnnnnnnnnnnnnngaggtctcaNNNNaaccaaggtccaaacggtgACGG 240
nnnnnnnnnnnnnnNNNNactctggagnnnnnnnnnnnnnnnnnctccagagtNNNNttggttccaggtttgccacTGCC

homology B (edit 1) | 5'-overhang(primer) | Separator(do not use) | 5'-overhang(primer)
C | C | target DNA Plasmid | PAM

BsaI BsaI

nnnnnnnnnnnnnnnnnnnnnnnnnnNNNNtgagacctcnnnnnnnnnnnnnnnnngaggtctcaNNNNnnnnnnnnnnn 320
nnnnnnnnnnnnnnnnnnnnnnnnnnNNNNactctggagnnnnnnnnnnnnnnnnnctccagagtNNNNnnnnnnnnnnn

homology A (edit 2) | Separator(do not use) | 5'-overhang(primer) | insert (edit 2)
5'-overhang(primer) | D | D

BsaI BsaI

nnnnnnnnnnnnnnNNNNtgagacctcnnnnnnnnnnngaggtctcaNNNNnnnnnnnnnnnnnnnnnnnnnnnnnnnnn 400
nnnnnnnnnnnnnnNNNNactctggagnnnnnnnnnnnctccagagtNNNNnnnnnnnnnnnnnnnnnnnnnnnnnnnnn

insert (edit 2) | 5'-overhang(primer) | homology B (edit 2)
E | 5'-overhang(primer) | E

BsaI ***End*** (414)

ncgcttgagacctc 3′ 414
ngcgaactctggag 5′

5'-overhang(primer)
homology B (edit 2)

	Feature		Location	Size (bp)	Type
	**source**		1 .. 187	187	source
	/organism	= unspecified			
	/mol_type	= genomic DNA			
✓	**5'-overhang(primer)**		1 .. 13	13	misc_feature
✓	**homology A (edit 1)**		14 .. 43	30	misc_feature
✓	**5'-overhang(primer)**		40 .. 52	13	misc_feature
✓	**A**		40 .. 43	4	misc_feature
	**source**		45 .. 50	6	source
	/organism	= unspecified			
	/mol_type	= genomic DNA			
	**source**		45 .. 50	6	source
	/organism	= unspecified			
	/mol_type	= genomic DNA			
✓	**Separator(do not use)**		53 .. 69	17	misc_feature
✓	**5'-overhang(primer)**		70 .. 82	13	misc_feature
	**source**		72 .. 77	6	source
	/organism	= unspecified			
	/mol_type	= genomic DNA			
✓	**A**		79 .. 82	4	misc_feature
✓	**insert (edit 1)**		83 .. 107	25	misc_feature
✓	**5'-overhang(primer)**		108 .. 120	13	misc_feature
✓	**B**		108 .. 111	4	misc_feature
	**source**		113 .. 118	6	source
	/organism	= unspecified			
	/mol_type	= genomic DNA			
✓	**5'-overhang(primer)**		132 .. 144	13	misc_feature
	**source**		134 .. 139	6	source
	/organism	= unspecified			
	/mol_type	= genomic DNA			
✓	**homology B (edit 1)**		141 .. 174	34	misc_feature
✓	**B**		141 .. 144	4	misc_feature
✓	**5'-overhang(primer)**		175 .. 187	13	misc_feature
	/note	= color: #ff6600; directio	/note="5'-overhang(primer)		
✓	**C**		175 .. 178	4	misc_feature
	**source**		175 .. 178	4	source
	/organism	= unspecified			
	/mol_type	= genomic DNA			
✓	**Separator(do not use)**		188 .. 204	17	misc_feature
	**source**		188 .. 204	17	source
	/organism	= unspecified			
	/mol_type	= genomic DNA			
	**source**		205 .. 414	210	source
	/organism	= unspecified			
	/mol_type	= genomic DNA			
✓	**5'-overhang(primer)**		205 .. 240	36	misc_feature
✓	**C**		214 .. 217	4	misc_feature

	Feature	Location	Size (bp)			Type
	**source**	214 .. 217	4			source
	/organism = unspecified					
	/mol_type = genomic DNA					
✓	**target DNA Plasmid**	218 .. 237	20			misc_feature
✓	**PAM**	238 .. 240	3			misc_feature
✓	**homology A (edit 2)**	241 .. 270	30			misc_feature
✓	**5'-overhang(primer)**	267 .. 279	13		←	misc_feature
✓	**D**	267 .. 270	4			misc_feature
	**source**	272 .. 277	6			source
	/organism = unspecified					
	/mol_type = genomic DNA					
	**source**	272 .. 277	6			source
	/organism = unspecified					
	/mol_type = genomic DNA					
✓	**Separator(do not use)**	280 .. 296	17			misc_feature
✓	**5'-overhang(primer)**	297 .. 309	13		→	misc_feature
	**source**	299 .. 304	6			source
	/organism = unspecified					
	/mol_type = genomic DNA					
✓	**D**	306 .. 309	4			misc_feature
✓	**insert (edit 2)**	310 .. 334	25			misc_feature
✓	**5'-overhang(primer)**	335 .. 347	13		←	misc_feature
✓	**E**	335 .. 338	4			misc_feature
	**source**	340 .. 345	6			source
	/organism = unspecified					
	/mol_type = genomic DNA					
✓	**5'-overhang(primer)**	359 .. 371	13		→	misc_feature
	**source**	361 .. 366	6			source
	/organism = unspecified					
	/mol_type = genomic DNA					
✓	**homology B (edit 2)**	368 .. 401	34			misc_feature
✓	**E**	368 .. 371	4			misc_feature
✓	**5'-overhang(primer)**	402 .. 414	13		←	misc_feature
	/note = color: #ff6600; directio	/note="5'-overhang(primer)				
