# Supplementary material for: A multifunctional system for genome editing and large-scale interspecies gene transfer
Source: Nat Commun. 2022 Jun 14;13:3430. doi: 10.1038/s41467-022-30843-1 (PMC9198041; doi:10.1038/s41467-022-30843-1)
Supplement: Supplementary file 5 — Source Data [file 41467_2022_30843_MOESM5_ESM.zip › Templates.zip!/Templates/homology_AIB_template.dna.pdf]

Sequence: homology_AIB_template.dna (Linear / 187 bp)
Enzymes: MoClo (6 of 646 total)
Features: 20 visible, 26 total

Unique Cutters **Bold**

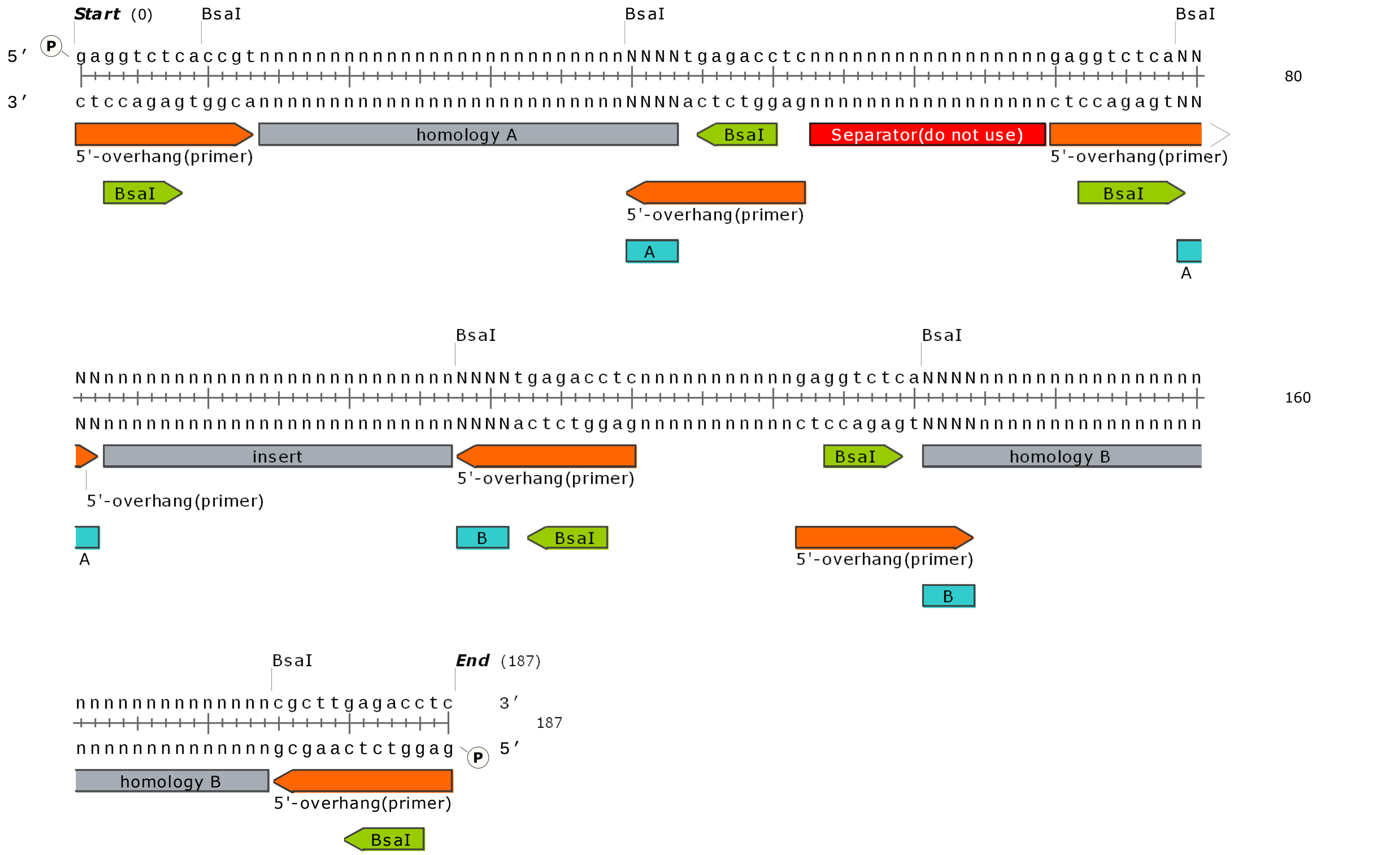

	Feature	Location	Size (bp)			Type
	**source**	1 .. 187	187		⟼	source
	/organism = unspecified					
	/mol_type = genomic DNA					
✓	**5'-overhang(primer)**	1 .. 13	13		→	misc_feature
✓	**BsaI**	3 .. 8	6		→	misc_feature
✓	**homology A**	14 .. 43	30		⟼	misc_feature
✓	**5'-overhang(primer)**	40 .. 52	13		←	misc_feature
✓	**A**	40 .. 43	4		⟼	misc_feature
✓	**BsaI**	45 .. 50	6		←	misc_feature
	**source**	45 .. 50	6		⟼	source
	/organism = unspecified					
	/mol_type = genomic DNA					
	**source**	45 .. 50	6		⟼	source
	/organism = unspecified					
	/mol_type = genomic DNA					
✓	**Separator(do not use)**	53 .. 69	17		⟼	misc_feature
✓	**5'-overhang(primer)**	70 .. 82	13		→	misc_feature
✓	**BsaI**	72 .. 79	8		→	misc_feature
	**source**	72 .. 77	6		⟼	source
	/organism = unspecified					
	/mol_type = genomic DNA					
✓	**A**	79 .. 82	4		⟼	misc_feature
✓	**insert**	83 .. 107	25		⟼	misc_feature
✓	**5'-overhang(primer)**	108 .. 120	13		←	misc_feature
✓	**B**	108 .. 111	4		⟼	misc_feature
✓	**BsaI**	113 .. 118	6		←	misc_feature
	**source**	113 .. 118	6		⟼	source
	/organism = unspecified					
	/mol_type = genomic DNA					
✓	**5'-overhang(primer)**	132 .. 144	13		→	misc_feature
✓	**BsaI**	134 .. 139	6		→	misc_feature
	**source**	134 .. 139	6		⟼	source
	/organism = unspecified					
	/mol_type = genomic DNA					
✓	**homology B**	141 .. 174	34		⟼	misc_feature
✓	**B**	141 .. 144	4		⟼	misc_feature
✓	**5'-overhang(primer)**	175 .. 187	13		←	misc_feature
	/note = color: #ff6600; directio	/note="5'-overhang(primer)				
✓	**BsaI**	180 .. 185	6		←	misc_feature
