# Supplementary material for: A multifunctional system for genome editing and large-scale interspecies gene transfer
Source: Nat Commun. 2022 Jun 14;13:3430. doi: 10.1038/s41467-022-30843-1 (PMC9198041; doi:10.1038/s41467-022-30843-1)
Supplement: Supplementary file 5 — Source Data [file 41467_2022_30843_MOESM5_ESM.zip › Templates.zip!/Templates/homology_A_template.dna.pdf]

Start (0) BsaI BsaI ***End*** (49)

5′ Ⓟ gaggtctcaccgtnnnnnnnnnnnnnnnnnnnnnnnggagtgagacctc 3′

49

3′ ctccagagtggcannnnnnnnnnnnnnnnnnnnnnncctcactctggag Ⓟ 5′

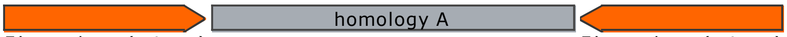

5'-overhang(primer) 5'-overhang(primer)

	Feature	Location	Size (bp)	Color	Direction	Type
	**source**	1 .. 49	49		⟷	source
	/organism = unspecified					
	/mol_type = genomic DNA					
✓	**5'-overhang(primer)**	1 .. 13	13		→	misc_feature
✓	**homology A**	14 .. 36	23		⟷	misc_feature
✓	**5'-overhang(primer)**	37 .. 49	13		←	misc_feature
