# Supplementary material for: A multifunctional system for genome editing and large-scale interspecies gene transfer
Source: Nat Commun. 2022 Jun 14;13:3430. doi: 10.1038/s41467-022-30843-1 (PMC9198041; doi:10.1038/s41467-022-30843-1)
Supplement: Supplementary file 5 — Source Data [file 41467_2022_30843_MOESM5_ESM.zip › Templates.zip!/Templates/homology_B_template.dna.pdf]

Sequence: homology_B_template.dna (Linear / 49 bp)
Enzymes: MoClo (6 of 646 total)
Features: 3 visible, 6 total

Unique Cutters **Bold**

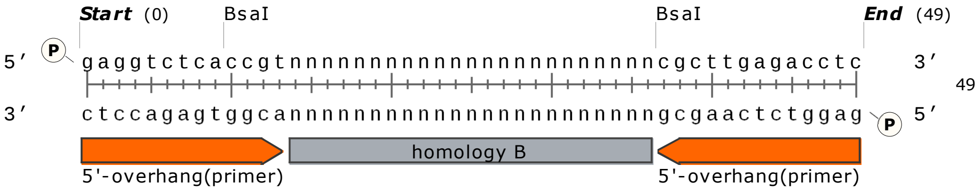

	Feature		Location	Size (bp)			Type
	**source**		1 .. 49	49			source
	/organism	= unspecified					
	/mol_type	= genomic DNA					
✓	**5'-overhang(primer)**		1 .. 13	13		→	misc_feature
✓	**homology B**		14 .. 36	23			misc_feature
✓	**5'-overhang(primer)**		37 .. 49	13		←	misc_feature
	**source**		37 .. 37	1			source
	/organism	= pKDsgRNA-ack					
	/mol_type	= other DNA					
	**source**		37 .. 37	1			source
	/organism	= pTKIP-cat					
	/mol_type	= other DNA					
