# Supplementary material for: A multifunctional system for genome editing and large-scale interspecies gene transfer
Source: Nat Commun. 2022 Jun 14;13:3430. doi: 10.1038/s41467-022-30843-1 (PMC9198041; doi:10.1038/s41467-022-30843-1)
Supplement: Supplementary file 5 — Source Data [file 41467_2022_30843_MOESM5_ESM.zip › Templates.zip!/Templates/insert_template.dna.pdf]

Start (0) BsaI BsaI ***End*** (42)

5′ (P) gaggtctcaggagnnnnnnnnnnnnnnnncgcttgagacctc 3′

42

3′ ctccagagtcctcnnnnnnnnnnnnnnnngcgaactctggag (P) 5′

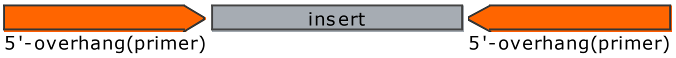

	Feature	Location	Size (bp)			Type
	**source**	1 .. 42	42	□	⟷	source
	/organism = unspecified					
	/mol_type = genomic DNA					
✓	**5'-overhang(primer)**	1 .. 13	13	■	→	misc_feature
✓	**insert**	14 .. 29	16	■	⟷	misc_feature
✓	**5'-overhang(primer)**	30 .. 42	13	■	←	misc_feature
