# Supplementary material for: A multifunctional system for genome editing and large-scale interspecies gene transfer
Source: Nat Commun. 2022 Jun 14;13:3430. doi: 10.1038/s41467-022-30843-1 (PMC9198041; doi:10.1038/s41467-022-30843-1)
Supplement: Supplementary file 5 — Source Data [file 41467_2022_30843_MOESM5_ESM.zip › Templates.zip!/Templates/target_2_template.dna.pdf]

Sequence: target_2_template.dna (Linear / 46 bp)
Enzymes: MoClo (6 of 646 total)
Features: 3 visible, 8 total

Unique Cutters **Bold**

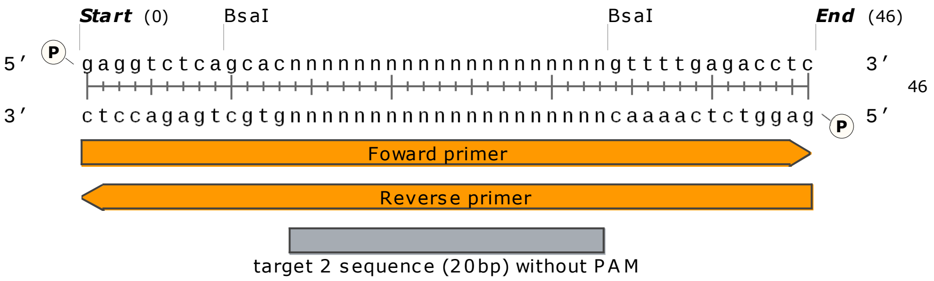

	Feature	Location	Size (bp)			Type
✓	**Foward primer**	1 .. 46	46		→	misc_feature
✓	**Reverse primer**	1 .. 46	46		←	misc_feature
	**source**	1 .. 46	46			source
	/organism = unspecified					
	/mol_type = genomic DNA					
✓	**target 2 sequence (20bp) without PAM**	14 .. 33	20			misc_feature
	**source**	34 .. 34	1			source
	/organism = pKDsgRNA-ack					
	/mol_type = other DNA					
	**source**	34 .. 34	1			source
	/organism = pTKIP-cat					
	/mol_type = other DNA					
	**source**	37 .. 37	1			source
	/organism = pKDsgRNA-ack					
	/mol_type = other DNA					
	**source**	37 .. 37	1			source
	/organism = pTKIP-cat					
	/mol_type = other DNA					
